# TFscope: systematic analysis of the sequence features involved in the binding preferences of transcription factors

**DOI:** 10.1186/s13059-024-03321-8

**Published:** 2024-07-10

**Authors:** Raphaël Romero, Christophe Menichelli, Christophe Vroland, Jean-Michel Marin, Sophie Lèbre, Charles-Henri Lecellier, Laurent Bréhélin

**Affiliations:** 1 LIRMM, Univ Montpellier, CNRS, Montpellier, France; 2 IMAG, Univ Montpellier, CNRS, Montpellier, France; 3 Institut de Génétique Moléculaire de Montpellier, University of Montpellier, CNRS, Montpellier, France; 4 AMIS, Université Paul-Valéry-Montpellier 3, Montpellier, France

## Abstract

Characterizing the binding preferences of transcription factors (TFs) in different cell types and conditions is key to understand how they orchestrate gene expression. Here, we develop TFscope, a machine learning approach that identifies sequence features explaining the binding differences observed between two ChIP-seq experiments targeting either the same TF in two conditions or two TFs with similar motifs (paralogous TFs). TFscope systematically investigates differences in the core motif, nucleotide environment and co-factor motifs, and provides the contribution of each key feature in the two experiments. TFscope was applied to > 305 ChIP-seq pairs, and several examples are discussed.

**Supplementary Information:**

The online version contains supplementary material available at 10.1186/s13059-024-03321-8.

## Introduction

Gene expression programming is the primary mechanism that controls the cellular phenotype and function. At the DNA level, transcription factors (TFs) are assumed to play a key role in this control. These proteins bind DNA sequence through specialized DNA-binding domains (DBDs) to enhance or repress the transcription of their target genes. DBDs preferentially bind to specific DNA sequences which are resumed in statistical models known as position weight matrices (PWMs) [58]. PWMs are usually obtained from dedicated probabilistic models—position probability matrices (PPMs)—which are available for many TFs in databases like JASPAR [20] and HOCOMOCO [34]. PWMs can be used to compute binding affinities and identify potential binding sites in genomes. However, contrary to bacterial DBDs which recognize sequences that often have sufficient information content to target particular genomic positions, most eukaryotic DBDs recognize short binding motifs (around 10 bp) that are not sufficient for specific targeting in the usually large (e.g., $10^9$ bp) eukaryotic genomes [61]. This purely statistical analysis has been corroborated by genome-wide studies based on sequencing approaches (ChIP-seq, ChIP-exo, CUT &RUN) that have been applied to hundreds of TFs to determine their binding profiles in various cell types and conditions [17]. These studies showed that most TFs only associate with a small subset of their potential genomic sites in vivo [57] and that the binding sites of a given TF often vary substantially between cell types and conditions [51]. Furthermore, as the number of DBD families in a genome is small relative to the number of TFs, TF paralogs from the same DBD family often share very similar binding motifs, yet they usually show distinct binding sites in vivo [3, 27, 33, 49]. Thus, it is now evident that DBD motifs, as modeled by PWMs, are not sufficient to completely determine TF binding in a specific cell or condition. On the other hand, several studies have revealed that a substantial number of the in vivo binding sites lack an obvious match with the known binding motif of the target TF [33, 57].

At this point, it is important to emphasize the strong links that exist between TF binding and histone marks [18]. Moreover, ChIP-seq experiments revealed that most TF binding sites (TFBSs) lie within highly accessible (i.e., nucleosome-depleted) DNA regions [54]. However, it remains unclear whether these chromatin states are a cause or a consequence of TF binding [26]. Moreover, recent approaches based on machine learning, and specifically convolutional neural networks (CNNs), have shown that transcription factor binding, but also gene expression, histone modifications, and DNase I-hypersensitive sites, can be predicted just from DNA sequences, often with surprisingly high accuracy [2, 30, 42, 56, 59, 63]. The good predictive performances of these approaches suggest that a large part of the instructions for gene regulation and TF binding are embedded in the DNA sequence.

Several mechanisms based on specific DNA features have thus been proposed to complement DBD motifs and explain how TFs target precise genome locations. The current view is that TF combinations underlie the specificity of eukaryotic gene expression regulation [14], with several TFs competing and collaborating to regulate common target genes. Multiple mechanisms can lead to TF cooperation [40, 43]. In its simplest form, cooperation involves direct TF-TF interactions before any DNA binding. Yet, cooperation can also be mediated through DNA, either with DNA providing additional stability to a TF-TF interaction [28] or without any direct protein-protein interaction, as in the pioneer/settler hierarchy described in Sherwood et al. [50] or in a non-hierarchical cooperative system such as the billboard model for enhancers [4, 39].

Besides TF combinations, other studies have investigated the role that the genomic environment around TFBS may have on the binding specificity, thereby revealing that some TFs have a preferential nucleotide content in the flanking positions of their core binding sites [15, 36]. Other studies have proposed that much larger regions containing repetitive sequences or multiple occurrences of low-affinity motifs may play an active role in TF binding [1, 12, 33]. Finally, another possibility that may be underestimated and that could also explain the binding specificity in certain cases is that, depending on the cell, condition, or TF paralog, the binding motif may actually differ, showing globally the same PWM to our eyes, but slightly changing on specific positions.

All of these mechanisms have been independently studied on specific cases, but a global computational approach is still lacking to investigate their role and relative importance in an automatized manner. The above-mentioned deep learning approaches are able to capture and combine the different sequence features involved, but identifying them via CNNs remains a difficult task [22, 32]. Although interesting methods are being developed to post-analyze CNN predictions and to identify single nucleotides and motifs (see e.g., [5, 32, 63]), disentangling all mechanisms/features captured by a CNN remains unreliable.

Here, we propose a machine learning approach called TFscope specially designed to explain the binding differences observed between two settings: two cell types, two treatments, or two paralogous TFs. In default mode, TFscope directly compares the two ChIP-seq data associated with the two settings by considering only regions unique to one or the other experiment. This strategy has two advantages. First, by focusing on the binding differences, there is an obviously gain in sensitivity for identifying the sequence features that best explain these differences. Second, we circumvent the common problem of the background definition which arises in all studies that aim to distinguish bound (foreground) versus unbound (background) genomic regions in a given cell type. While the definition of the foreground is straightforward, the definition of the background is often much more challenging and highly influences the results and conclusions (see for example references [41, 59, 60, 62] for interesting considerations about the background issue).

Given two ChIP-seq data, our method systematically investigates the importance of (i) the core motif, (ii) the genomic environment, and (iii) the cooperative TFs for predicting binding differences between two data. TFscope is based on three different modules that capture these three levels of information. The first module captures potential differences in the core motif. This module is based on a new method that learns discriminative PWMs. Note that that well known approaches such as DREME/STREME [6], DAMO [46], and Homer [24] have already been proposed for this task. These methods are, however, designed for a slightly different and computationally more complex problem that is not exactly the same as ours. They thus rely on sub-optimal heuristics while an optimal algorithm exists for our problem. The second module captures the nucleotidic environment in the form of short k-mers (2–4 bps) enriched in specific regions around the core motif and is based on our DExTER method [38]. The third module is a refinement of our TFcoop method that identifies co-factors and TF combinations involved in the binding of a target TF [56]. In a final step, these data are jointly used in a global predictive model that can quantify the relative importance of each information item for the problem at hand. Hence, in contrast to CNN based methods [5, 62], our approach completely controls the predictive features input into the model. This allows us to easily measure the importance of each feature by computing the loss of accuracy induced by its withdrawal from the model, which is very challenging to do with classical CNN approaches.

We applied TFscope to more than 350 ChIP-seq pairs targeting either a common TF in two different cell types or treatments or two paralogous TFs in the same cell type. Our results showed that classification is very often accurate and that the most important sequence features greatly vary depending on the TFs and conditions. For TFs in different cell types or with different treatments, either co-factors or the nucleotidic environment often explains most of the binding-site differences. Moreover, when co-factors are involved, which is the most frequent case, their position on the DNA relative to the core motif is also important. On the contrary, for paralogous TFs, the core motif seems to be the most important factor in our experiments. Although the motifs of paralogous TFs show very similar PWMs, subtle differences at specific positions explain most of the binding differences.

## Results

### TFscope overview

**Fig. 1:**
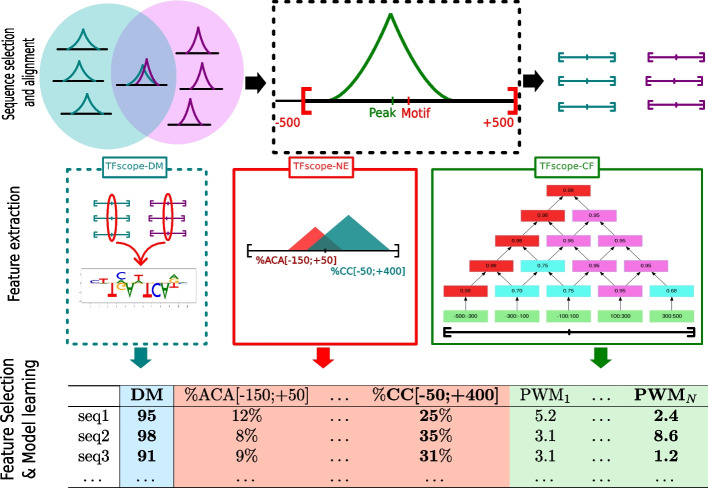
The TFscope approach. In the first step (sequence selection and alignment), the set of ChIP-seq peaks to be compared are selected. In a classical analysis, peaks in the intersection (i.e., associated with both ChIP-seq experiments) are removed, and the peaks in the two complementary sets are selected for comparison. Alternatively, users can choose to compare one of the complementary sets vs. the intersection set. Next, the most likely TFBSs of the selected peaks are identified and used to extract the 1 Kb sequences centered on these sites. If the motif of the target TF is unknown, this step can be skipped and sequences remain centered on the peak summit. All sequences are then used for the second step (feature extraction). Three dedicated modules extract three kinds of sequence features that can be useful for discriminating the two classes. The TFscope-DM module learns a new PWM that discriminates the sequences solely on the basis of the core motif (if the target motif is unknown this module is also skipped). The TFscope-NE module searches for specific nucleotidic environments (i.e., frequency of specific k-mers in specific regions) that are different in the two classes. The TFscope-CF module searches for binding sites of specific co-factors whose presence in specific regions differs between the two classes. All of these features (variables) are then gathered into a long table, and a logistic model (Expression (1)) is learned on the basis of these data (Feature selection and model learning step). A special penalty function (LASSO) is used during training, for selecting only the best variables in the model (in bold in the table)

TFscope aims to identify the sequence features responsible for the binding differences observed between two ChIP-seq experiments. Typically, TFscope can be used to identify differences between two experiments targeting the same TF in different cell types or conditions, or two experiments targeting two paralogous TFs that share similar motifs. TFscope takes as input two sets of ChIP-seq peaks corresponding to the two ChIP-seq experiments and then runs the three steps illustrated in Fig. 1: sequence selection and alignment, feature extraction, and model learning. In the sequence alignment step, TFscope first selects the two sets of peaks to analyze. By default, the comparison focus on the peaks that are unique to the first vs. the second experiment (see the “Material and methods” section). Alternatively, users can choose to compare the peaks unique either to the first or the second experiment *vs.* the intersection set. A last possibility is to use a dedicated differential peak caller to identify differential peaks between the two experiments [16]. Then, TFscope identifies the most likely binding site using a strategy similar to Centrimo [8] and UniBind [21] and parses the sequence around the peak summit with the PWM associated with the target TF (if several versions of the motif are available or if the analysis involves two paralogous TFs with similar motifs, the most discriminative PWM is chosen to scan the two sets of sequences; see Material and Methods). The FIMO tool [23] is used for this analysis, and the position with the highest PWM score serves as an anchor point to extract the 1 Kb long sequence centered around this position. At the end of the alignment step, we get two classes of sequences centered on the most likely TFBSs of the input ChIP-seq peaks. Sequences with no occurrence of the motif around the peak summit are discarded. Alternatively, if the motif of the target TF is unknown, or if the user prefers to keep all sequences, the search of the most likely TFBS is skipped, and sequences remain centered on the peak summits.

TFscope then runs the three modules detailed below to extract three kinds of sequence features that are discriminative of the two sequence classes (feature-extraction step). The first module (TFscope-DM) learns a new PWM of the core motif. This PWM differs from the original PWM used to parse the sequence, as it focuses on potential core motif differences that may exist between the two sequence sets. This module returns a single variable $\text {DM}(s)$, which is the score of the new PWM on each sequence *s*. Note that this module is omitted if the motif of the target TF is unknown. The second module (TFscope-NE) searches for pairs of (k-mer,region) for which the frequency of the k-mer in the defined region is different between the two sets of sequences. For example, the frequency of the 3-mer ACA in region $[-150:+500]$ (with 0 being the anchor point of the sequences) may be globally higher in sequences of the first class than in those of the second class. The idea is to capture nucleotidic environment differences that may exist between the two classes. We use for this module a slight modification of the DExTER method recently proposed to identify long regulatory elements [38]. This module returns a potentially large set of $\text {NE}_i(s)$ variables that corresponds to the frequency of *i*th k-mer in the associated *i*th region for each sequence *s*. The third module (TFscope-CF) uses a library of PWMs (in the experiments below the JASPAR2020 library was used [20]) and searches for pairs of (PWM,region) for which the PWM score in the identified region is different between the two sets of sequences (see below). The idea is to identify all co-factors of the target TF whose binding sites differ between the two classes: either because these binding sites are in majority present in one class and not the other or because the locations of these binding sites differ between the two classes. This third module returns a set of variables $\text {CF}_j(s)$ that corresponds to the score of the *j*th PWM in the identified *j*th region for each sequence *s*.

All variables are then integrated into a global model that aims to predict if a sequence belongs to the first or the second class (learning step). We used a logistic regression model:

1 $$\begin{aligned} P(1|s) = S\left( a\cdot \text {DM}(s) + \sum \limits _i b_i\cdot \text {NE}_i(s) + \sum \limits _j c_j\cdot \text {CF}_j(s)\right) , \end{aligned}$$

where *P*(1|*s*) is the probability that sequence *s* belongs to the first class, *S* is the sigmoid function, $\text {DM}(s)$ is the score of the discriminative motif for sequence *s*, $\text {NE}_i(s)$ is the value of the *i*th nucleotidic-environment variable for sequence *s*, $\text {CF}_j(s)$ is the value of the *j*th co-factor variable for sequence *s*, and *a*, $b_i$ and $c_j$ are the regression coefficients which constitute the model parameters. Because the set of variables identified by the last two modules is usually large and the variables are often correlated, the model is trained with a LASSO penalty function [55] that selects the most relevant variables—i.e., many regression coefficients (*a*, $b_i$ and $c_j$) are set at zero. We use for this the glmnet package in R. Note that the right amount of penalization is automatically deduced from the data by glmnet using an internal cross-validation procedure. Finally, once a model has been trained, its accuracy is evaluated by computing the area under the ROC (AUROC) on several hundred sequences. To avoid any bias, this is done on a set of sequences that have not been used in the previous steps ($70\%$ sequences are used for training, and 30$\%$ for the test).

#### TFscope-DM: identification of differences in the core motif

The first TFscope module learns a new discriminative PWM. Recall that at the end of the alignment step, the most likely binding site of each ChIP-seq peak has been identified with the JASPAR PWM associated with the TF, and all sequences are aligned on these sites. If several versions of the PWM are available, the most discriminative PWM is used (see Material and Methods). We then extract the *K*-length sub-sequence corresponding to the occurrence of the motif in each sequence (with *K* being the size of the PWM). The first module aims to learn a new PWM that could discriminate these two sets of *K*-length sequences. First, each sequence *s* is one-hot encoded in a $K\times 4$ matrix **s**. Then a logistic model with $K\times 4$ parameters is learned to discriminate the two sequence classes:

2 $$\begin{aligned} P(1|s) = S\left( \sum \limits _{k=1}^K \sum \limits _{j=1}^4 a_{k,j} \cdot \textbf{s}_{k,j}\right) , \end{aligned}$$

where *P*(1|*s*) is the probability that sub-sequence *s* belong to the first class, *S* is the sigmoid function, $\textbf{s}_{k,j}$ is the entry of the one-hot matrix **s** indicating whether the *k*th nucleotide of sequence *s* corresponds to the *j*th nucleotide of $\{A,T,G,C\}$ or not, and $a_{k,j}$ is the regression coefficients of the model.

Once this model has been learned, it can be used to predict if a sequence belongs to the first or second class. The sigmoid function being monotonically increasing, this can be done easily by computing the linear function inside the parenthesis of Expression (2) and using the result as a score reflecting the likelihood of class 1. Interestingly, this score function has exactly the same form as that used to compute a score with a PWM. Consequently, the logistic model of Expression (2) is strictly speaking a regular PWM with parameters $a_{k,j}$. The interest of learning a PWM in this way is twofold. First, we take advantage of all the algorithmic and theory developed for logistic regression. Most notably, as the likelihood function of a logistic model is convex, we are guaranteed that the learned model is optimal, which means that the inferred discriminative PWM is the best PWM for our problem. This is a major difference from approaches previously proposed to learn a discriminative PWM, such as DAMO [46] or STREME [6]. The reason for this is that these approaches do not exactly address the same problem as ours: they do not search for a PWM that discriminates two sets of sequences that are perfectly aligned and of the same length as the PWM. Instead, they take as input two sets of sequences that are usually much longer than the PWM, and their goal is to identify a motif whose presence can be used to discriminate the two sets, i.e., a problem that is known to be NP-hard [37]. Hence, these approaches rely on heuristics and do not warrant returning the best PWM for our problem (see the “Discussion” section for more details on these differences). The second advantage of learning a PWM via a logistic regression approach is that a LASSO penalty may be included in the optimization procedure in order to obtain a model with fewer variables [55] (see the “Material and methods” section). In practice, this means that many $a_{k,j}$ parameters are set at zero and hence that the resulting PWM is simpler and easier to interpret.

**Fig. 2:**
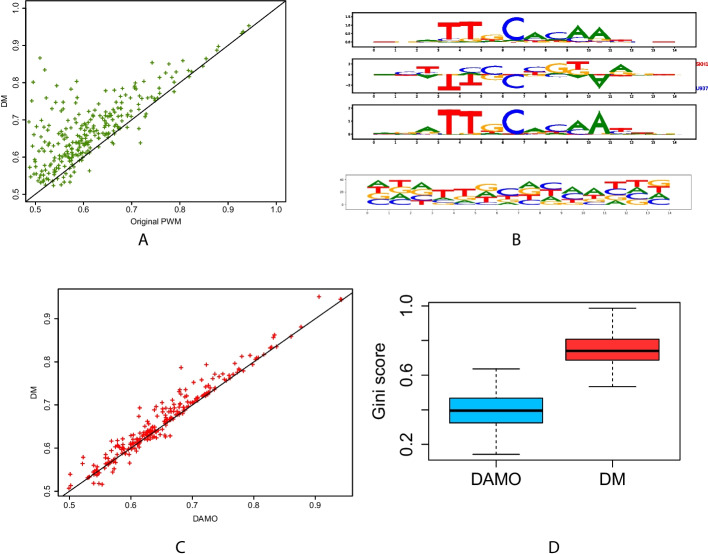
TFscope learns discriminative and informative motifs. **A** AUROCs achieved by the TFscope PWMs *vs.* original PWMs on the 272 experiments. **B** The first three logos represent the PWM learned by TFscope for discriminating CEBPA binding between U937 and SKH1 cell types in between the two PPMs estimated on these two cell types. The bottom logo represents the discriminative PWM learned by DAMO on the same training set. **C** AUROCs achieved by the TFscope PWMs *vs.* DAMO PWMs on the 272 experiments. **D** Gini score of the PWMs learned by TFscope and DAMO. The higher the Gini score, the simpler the model

Note that, like DAMO [46], the PWMs output by our method are not obtained from position probability matrices (PPMs), i.e., the probabilistic models that are often associated with PWMs. This avoids the constraints attached to PPMs (see section Discussion and the work of Ruan and Stormo [45] for further details), but this also impedes representing PWMs with the classical logo graphics based on information theory [47]. Instead, our PWMs are represented by “mirror-logos” such as that in Fig. 2B (middle). These logos provide the sign of the parameters, which allows us to easily distinguish nucleotides that are more present in sequences of one or the other class.

#### TFscope-NE: identification of differences in nucleotidic environment

The second TFscope module extracts features related to the nucleotidic environment around the core binding motif. More precisely, this module constructs variables defined by a pair (kmer,region) such that the frequency of the identified k-mer in the identified region is, on average, different in the two classes. We used for this a slight modification of the DExTER method initially proposed to identify pairs of (kmer,region) whose values are correlated with an expression signal. The DExTER optimization function was modified to return variables correlated with classes rather than with expression signals (see the “Material and methods” section). The TFscope-NE module explores short k-mers up to length 4. To prevent this module from capturing information related to the core-motif, this motif is masked before running the TFscope-NE analysis.

#### TFscope-CF: identification of differences in co-factor combinations

The third TFscope module extracts features related to co-factors. This module constructs variables defined by a pair (PWM,region) such that the score of the PWM in the identified region is, on average, different between the two classes. For example, we may observe that sequences of the first class often have a potential binding site for a specific TF in region [-250,0] upstream of the binding site of the target TF, while the sequences of the second class do not have these potential binding sites. Hence, the goal of this module is to identify, for each PWM of the library, a specific region of sequences in which the scores of this PWM are higher in one class than in the other one.

Sequences are first segmented in bins of the same size. We used 13 bins in the following experiments. The number of bins impacts the precision of the approach but also the computing time for the analysis. For each PWM, TFscope scans all sequences with FIMO [23], and the best score achieved on each bin of each sequence is stored. Then, TFscope searches the region of consecutive bins for which the PWM gets the most different scores depending on the class of the sequences. A lattice structure is used for this exploration (see Fig. 1 and details in the ”Material and methods” section). For each PWM of the library, TFscope-CF selects the region that shows the greatest differences and returns a variable corresponding to this PWM and region. As for TFscope-NE, the core-motif is masked before running the analysis.

### Analysis of the cellular specificities of 272 ChIP-seq pairs

We first sought to apply TFscope to identify binding site differences of TFs in different cell types using a selection of 272 pairs of ChIP-seq experiments downloaded from the GTRD database [31]. Data were filtered using the UniBind *p*-value score [21] to minimize the effects linked to technical issues or indirect binding. In UniBind, the authors studied the distance between the ChIP-seq peaks and the position of the most likely binding site (inferred with the PFM associated with the studied TF). They showed that this binding site is sometimes far from the ChIP-seq peak and that the peak could be a false positive. A dedicated method named ChIP-eat determines genomic boundaries inside which the binding sites are likely true positives and provides a *p*-value measuring peak enrichment in these boundaries. We used this *p*-value to remove ChIP-seq experiments that could be affected by technical issues and indirect binding. Moreover, for this analysis, we only selected pairs of experiments that showed strong binding site differences according to the Jaccard’s distance (see the “Material and methods” section). The 272 pairs were chosen to provide a wide view of the ChIP-seq data in GTRD, i.e., pairs that were too close to another already selected pair were discarded (see the pair selection procedure in the “Material and methods” section). These 272 pairs involve a total of 86 different TFs and 168 cell types (see Additional file 1: Fig. S1A for statistics about the number of pairs targeting each TF). The distribution of Jaccard indexes of all pairs of ChIP-seq with Unibind *p*-value $<10^{-2}$ is shown in Additional file 1: Fig. S1B. For comparison, the figure also reports the Jaccard index of ChIP-seq pairs targeting the same TF in the same cell type, but originating from different studies. As we can see, ChIP-seq pairs from different cell types are usually much more different than in experiments with the same cell type. Hence, in these experiments, many of the differences observed between cell types are likely due to the cellular specificity rather than the technical artifacts. Moreover, a low Jaccard index also means that the number of common peaks is small in proportion, hence comparing unique peaks makes sense for these analyses. The Jaccard index distribution of the 272 selected ChIP-seq pairs is shown in Additional file 1: Fig. S1C. Among the peaks of these experiments, a very low number ($<2\%$) do not have the target motif (Additional file 1: Fig. S1D) and were discarded.

### TFscope learns both discriminative and informative core motifs

We first assessed the TFscope ability to identify core motif differences in the ChIP-seq experiment pairs. In this analysis, we only used the score function of the learned PWM (Expression (2)) to discriminate the two cell types. For comparison, we also used the score of the original PWM on this problem. Accuracies were measured by AUROC on an independent set of sequences (see Fig. 2A). If several versions of the original PWM were available, we used the version that provides the best AUROC. As we can see, the new PWM outperforms the original PWM most of the time. Moreover, we can also observe that for some of the 272 experiment pairs, the core motif is sufficient to differentiate the two cell types with high accuracy (AUROC $>75\%$ for 49 experiments). As already discussed, the discriminative PWM differs from the original PWM as it specifically models the differences while removing features common to the two classes. The “mirror-logo” representation summarizes these differences and shows which features are associated with which cell type. To help interpret this logo, TFscope also outputs the PPMs built from the sequences associated with the two cell types. We used for this the K-length sub-sequences that were used by TFscope-DM to learn the discriminative PWM and independently estimated two PPMs from these two sequence sets simply by counting the frequency of each nucleotide at each position. For example, the first three logos on Fig. 2B show the mirror logo of the discriminative PWM learned by TFscope for discriminating CEBPA binding sites between the SKH1 and U937 cell types, in between the two PPMs associated with these cell types. Note here that the canonical CEBPA motif is more often associated with ChIP-seq peaks collected in U937 than in SKH1, although both cell types show very similar motifs. The mirror logo indicates for example that the T nucleotides at positions 3 and 4 are more often missing in the SKH1 sequences than in the U937 sequences.

As an alternative approach, we compared the accuracy of the discriminative PWM to that of the PPMs built directly from the sequences associated with the two cell types. For this, the two PPMs were transformed in PWMs and combined in a logistic model trained to determine if a sequence belongs to the first or the second cell types on the basis of a linear combination of the two PWMs (see the “Material and methods” section). As shown in Additional file 1: Fig. S2A, the PWM learned by TFscope outperforms this two-PWMs approach most of the time and especially at AUC$>70\%$, illustrating that discriminative PWMs capture sequence features that are not captured when estimating PWMs independently on each cell type. We also used the same approach using PWMs learned with Homer [24] instead of the PPMs independently estimated on the two cell types, and observed that the TFscope PWM also outperforms the two-Homer-PWM approach on these experiments (Additional file 1: Fig. S2B and the “Material and methods” section for details).

We next sought to compare these results to those obtained with another method designed to optimize PWMs using a discriminative approach. We used the DAMO approach for this comparison, as it is one of the rare methods that do not rely on PPM to learn a PWM. Recall that DAMO, like other classical approaches to learn PWMs, was not designed to exactly address the same problem as ours. Indeed, DAMO usually takes as input sequences that are not aligned and that are much longer than the target PWM. Nevertheless, it could also be used on our simpler problem. However, as illustrated in Fig. 2C, it does not achieve the same accuracy as TFscope on this problem, which was somewhat expected as the logistic classifier used by TFscope theoretically returns the most discriminative PWM.

Another striking fact that emerged when we compared the discriminative motifs learned by DAMO to those of TFscope was that the DAMO motifs appear much more complex, with many positions without clear preferences (bottom logo on Fig. 2B). On the contrary, thanks to the LASSO penalty used for learning, the TFscope motif is easier to interpret, with many positions set at zero. This aspect was assessed systematically on the 272 experiments using a score function based on the Gini coefficient for measuring the motif simplicity (see the “Material and methods” section). As illustrated in Fig. 2D, TFscope motifs have higher Gini coefficient, and are thus simpler and easier to interpret than their DAMO counterpart.

Finally, we observed that increasing the size of the PWM on both sides slightly improves the AUROC of the model, especially until 4 nucleotides (see Additional file 1: Fig. S2C). After 4 nucleotides, further increasing the size of the flanking regions still slightly increases the AUROC, but the gain is likely due to PWM capturing part of the large nucleotidic environment, something specifically captured by the TFscope-NE module. Hence, to facilitate the model interpretation, in the following we only used the discriminative PWM with 4 flanking nucleotides on both sides (denoted as DM+8).

### TFscope provides meaningful information about co-factors

**Fig. 3:**
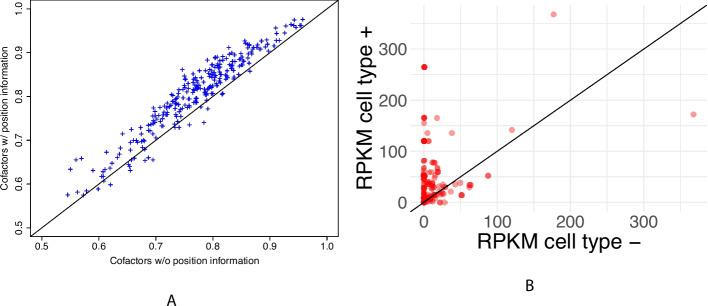
TFscope provides meaningful information about co-factors. **A** Position of co-factors helps for predicting the cell-specificity. This plot reports AUROCs achieved by TFscope models using two different scores for co-factors. On the *x*-axis, the score of a co-factor on a sequence is the best achieved at any position of the sequence, i.e., the co-factor position is not considered in the score computation. On the *y*-axis, the score of each co-factor is only computed on a specific region identified by the TFscope-CF module to be the most informative for this co-factor. **B** Gene expression level (RPKM) of co-factors identified by TFscope. Cell type $+$ (resp. −) is the cell type where sequences have the highest (resp. lowest) scores for the PWM associated with the co-factor

We next sought to investigate the information gained by the position of the binding sites of potential co-factors for cell type prediction. For this, we used a simplification of the model of Expression (1) which only uses the core motif and the co-factor variables for the prediction—i.e., the $\text {NE}_i$ variables capturing the nucleotidic environment were removed from the model. The accuracy of this model was compared to that of a similar model that also uses the score of potential co-factors, but without integrating the position information. This model, which strongly resembles the TFcoop approach we previously proposed [56], simply uses the best score achieved by the different PWMs in the whole sequence. Hence, the predictive variables of this model are the best scores achieved at any position in the sequence, while in Expression (1) TFscope uses the best score achieved in a specific region identified as the most informative for each co-factor. While the two models have exactly the same number of parameters (i.e., the number of PWMs in the PWM library), the TFscope variables greatly increase the accuracy of the approach (Fig. 3A), illustrating that the position of co-factors relative to the considered TFBS also carry important information. Note that, as we will see hereafter, TFscope provides a graphical representation of all identified co-factors, and position information can be easily retrieved.

Finally, we attempted to assess the relevance of the co-factors identified by TFscope. We thus selected all experiments comparing cell-line pairs involving HepG2, K562, MCF7, GM12878, MCF10A, or IMR90. These cell lines were chosen because they were among the most represented cell lines in the 272 experiments. This involves a total of 19 experiments. RNA-seq data measuring gene expression in the same cell lines were downloaded from ENCODE. Next, for each of the 19 experiments, we extracted the 15 most important variables selected by the model (see the “Material and methods” section) and identified the co-factors present among these 15 variables (this involves a total of 203 co-factors for the 19 experiments). For each co-factor, we then compared its gene expression level (RPKM) in the cell line where it had been identified as associated with the target TF (denoted as cell type $+$), and in the other cell line (cell type −). As we can see in Fig. 3B, the gene expression level of the co-factor is usually higher in cell type $+$ than in cell type − (149 vs. 45, binomial test *p*-value $3.e^{-14}$). Similarly, for 79 co-factors, the gene expression level is null in cell type − (RPKM$<1$) and non-null in cell type $+$ (RPKM$>1$), while the opposite is true for only 4 co-factors (binomial test *p*-value $< 2.e^{-16}$). Hence, very often, the differential presence of binding motifs between the two cell types is corroborated by the difference of expression of the identified co-factors.

### TFscope assesses the relative importance of each sequence feature

**Fig. 4:**
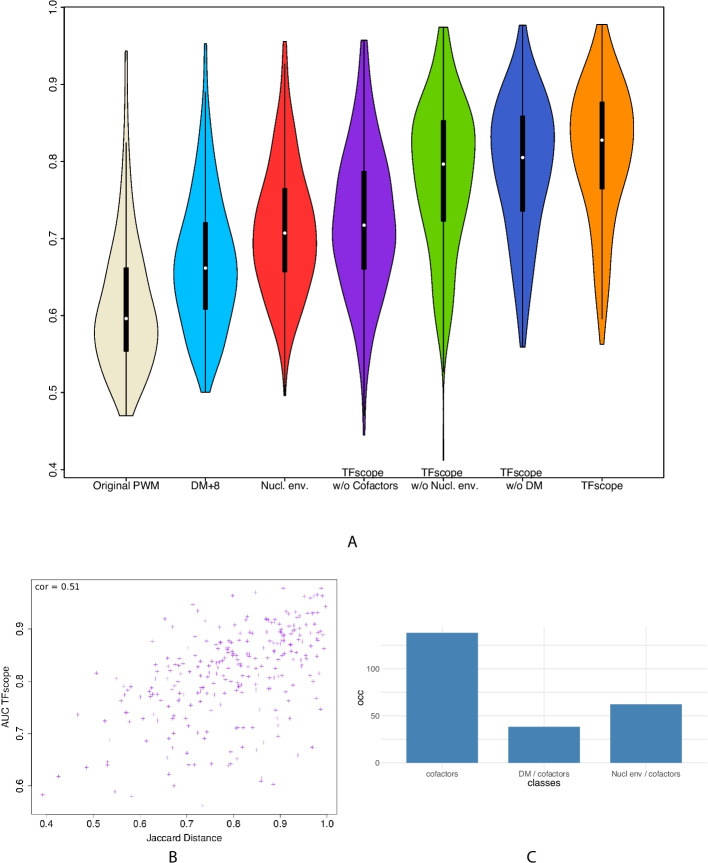
Accuracy achieved by TFscope for discriminating binding sites of different cell types. **A** Distribution of AUROCs achieved by TFscope and several alternative models for discriminating binding sites of one TF in two different cell types. **B** Link between TFscope accuracy and the similarity of ChIP-seq peaks in the two cell types. ChIP-seq experiments that have a high proportion of peaks in common have a low Jaccard distance (Jaccard distance = 1 - Jaccard index). **C** Distribution of TFscope models according to the most discriminative features: mainly co-factors, discriminative motif $+$ co-factors, or nucleotidic environment $+$ co-factors

We next ran TFscope with the full model of Expression (1) on the 272 pairs of experiments and compared its accuracy (AUROC) to that of different alternatives: the original PWM only, the discriminative PWM only, and three incomplete TFscope models that only use two of the three kinds of genomic information. These incomplete models were obtained by taking the full TFscope model trained with all variables and by setting at zero either the $\text {DM}$ variable (model TFscope w/o core motif information) or the $\text {NE}_i$ variables (TFscope w/o nucleotidic environment information) or the $\text {CF}_j$ variables (TFscope w/o co-factor information). Figure 4A reports the accuracy achieved by all these models. As we can see, the full TFscope model successfully integrates the three kinds of genomic information and outperforms the alternative models. Note also that the accuracy is often good, with a median AUROC above $80\%$. Moreover, there is a strong link between the accuracy of the approach and the Jaccard distance between the ChIP-seq peaks in the two cell types (Pearson *r *= 0.51; see Fig. 4B), i.e., experiments with a low proportion of ChIP-seq peaks shared by the two cell types often have good accuracy (remember that these peaks are removed before the analyses). In other words, when the two ChIP-seq experiments are really different, TFscope accurately predicts these differences. For the sake of generality, we also ran TFscope using HOCOMOCO PPMs instead of JASPAR PPMs on a random selection of 10 experiments and observed very similar accuracy (Additional file 1: Fig. S3.A).

**Fig. 5:**
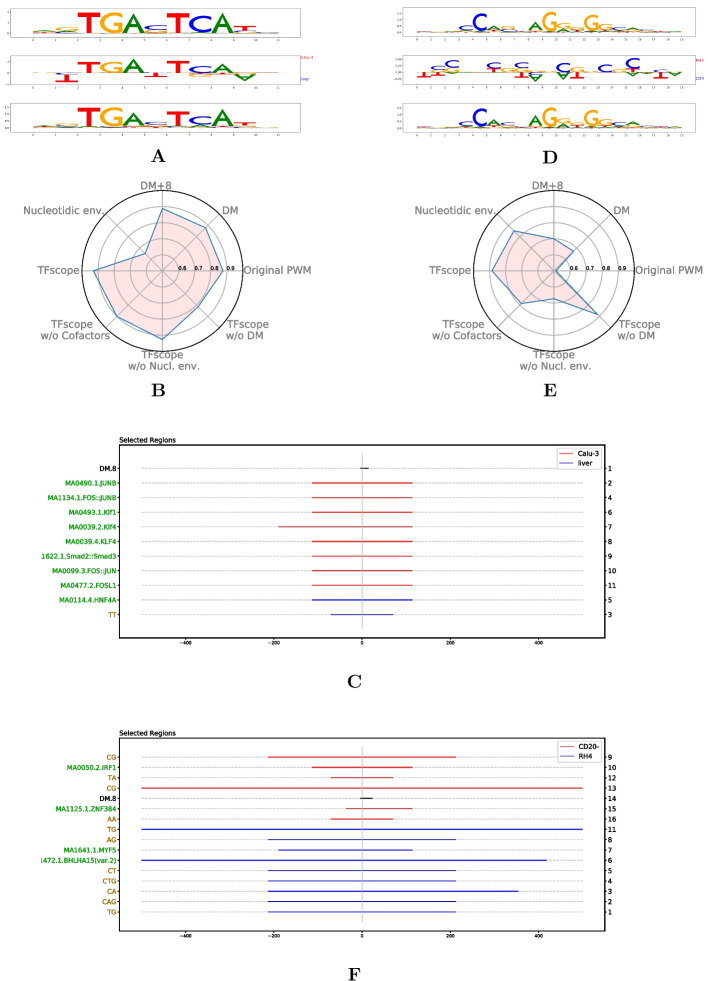
Core motif, nucleotidic environment and co-factors together determine cell specificity. **A–C** PWM logos, radar plot, and location of the most important variables in the JUND comparison between liver and lung carcinoma. **D–F** Discriminative PWM, radar plot, and location of the most important variables in the CTCF comparison between B lymphocyte and rhabdomyosarcoma. In the PWM logos (**A** and** D**) the discriminative PWM is in between the two PPM logos estimated on the two cell types. Radar plots (**B** and **E**) summarize the AUROC achieved by TFscope and several alternative models. Location plots (**C** and** F**) provide the identity and location of the most important variables (black: DM; green: co-factors; brown: nucleotidic environment). The numbers on the right indicate the variable ranks, from the most important (rank 1) to the least important. The color of segments indicates the cell type associated with each variable

These good performances legitimate the use of TFscope to investigate the relative importance of each kind of genomic information in the different comparisons. For this, in addition to the logo of the discriminative PWM, TFscope outputs a radar plot that summarizes the accuracy of the different models and alternatives and a location plot that summarizes the position of the most important variables of the model (see the “Material and methods” section). For example, Fig. 5B reports the radar plot obtained when analyzing the binding differences of TF JUND between liver and lung carcinoma. For this experiment, the core motif is clearly the most discriminant information (Fig. 5A), since removing this information lead to the largest drop in AUROC. Besides, peaks detected in lung harbor additional AP-1 motifs around the core motif (Fig. 5C). JunD belongs to the AP-1 family of dimeric TFs, which associate members of the Jun (c-Jun, JunB and JunD) and Fos (c-Fos, FosB, Fra-1/Fosl1 and Fra-2/Fosl2) families. The canonical view is that Jun family members can homodimerize, while Fos family members must, at physiological concentrations, heterodimerize with one of the Jun proteins to bind DNA. The c-Fos proteins homodimerize only when overexpressed [53]. Importantly, Fos:Jun heterodimers have a stronger affinity for DNA than the Jun:Jun homodimers [9]. According to various expression data listed in the EBI Expression Atlas (https://www.ebi.ac.uk/gxa/home), Fos TFs are less expressed in liver than in lung. Thus, the JunD binding preferences observed in liver vs. lung might merely be explained by the expression of Fos TFs: because the probability of forming Fos:Jun heterodimers is greater in lung than in liver, JunD will bind DNA with a higher affinity in lung than in liver. For comparison, the discriminative motif and the radar plot of the CTCF experiment between CD20 and RH4 are shown in Fig. 5D–E. Here, the most discriminative information seems to be the nucleotidic environment. The location plot provides additional information (Fig. 5F). We can see that CD20 favors A/T rich environment in the vicinity of the binding motif ($\sim +/-100$ bp around the motif), and C/G nucleotides in the larger surrounding region ($+/-500$ bp). On the contrary, RH4 prefers a nucleotide environment rich in TG and CA dinucleotides. All results obtained on the 272 experiments are available at https://gite.lirmm.fr/rromero/tfscope/-/tree/main/results.

Finally, in an attempt to provide a broad picture of the genomic strategies involved in the control of binding differences between cell types, we ran a K-means clustering on the importance profiles inferred by TFscope. More precisely, all 272 experiments were described by a vector of length 3 obtained by subtracting the AUROC of TFscope w/o DM, TFscope w/o NE, and TFscope w/o CF from that of the full TFscope model. Each experiment was then represented by three values representing the three AUROC losses associated with the three kinds of information. We then ran several K-means clustering with different numbers of classes to study the distribution of the experiments. A plot of cluster variance according to the number of classes *k* suggests that the most common control-strategies can be broadly classified into 3 or 4 classes (Additional file 1: Fig. S3.B). We thus set $k=3$ for simplicity, and got the following 3 classes: (1) one class where the co-factors are clearly the most important feature, (2) one class where DM (major) along with the co-factors (minor) are the most important features, and (3) one class where the nucleotidic environment (major) along with the co-factors (minor) are the most important features. Figure 4C reports the distribution of the 272 models in these 3 classes, thus highlighting the fact that the co-factors are by far the most common mechanism involved in the binding differences between cell types. Interestingly, for 32 out of 248 experiments with an AUROC $>70\%$ (13$\%$), the PWM of the target TF is also present among the co-factors, which means that the TF often has additional binding motifs around the core binding site identified as the most likely TFBS of a sequence and that these additional binding motifs are more present in one of the two cell types.

### TFscope correctly handles repeat elements

Repeat elements, and notably short tandem repeats, may play an important role in TF binding [1, 12, 25, 33]. These sequences are handled in TFscope via the TFscope-EN module. According to the above experiments, in a majority of cases ($58\%$), co-factors are the most influential feature, while the nucleotide environment is the primary predictor in $26\%$ of experiments. We thus sought to study how TFscope compares to a model that would predict cell specific binding just on the basis of known repetitive sequences. We thus used the 20 classes of repetitive elements listed in RepeatMasker. Specifically, the coordinates of each input sequence were intersected with those of RepeatMasker classes. Each sequence was then described by the presence/absence of 20 different repeat classes. For each ChIP-seq comparison, this classifier was evaluated on the same test set as that used for TFscope, and the AUROC was calculated. For most comparisons, AUROC was $< 60\%$ (Additional file 1: Fig. S4), thereby suggesting that, in most cases, repeats classes, as opposed to the nucleotidic environment measured by TFscope, play a modest role in discriminating cell type specific TF binding. One noticeable case was NR3C1 in HepG2 vs. MCF10A, where we found that the repeat-based classifier yields an AUROC around $87\%$. We looked at enriched repeat classes in these two ChIP-seq experiments and found that NR3C1 peaks were enriched in satellites in HepG2, and in simple repeats in MCF10A (Additional file 1: Table S1), mostly (TC)n and (CT)n. These findings are in agreement with the important features identified by TFscope, which highlights the role of regions rich in 3-mer CTC and of ZNF263 and PRDM1 binding motifs (two CT/TC-rich motifs), in turn illustrating that TFscope is able to capture the potential impact of repetitive elements on TF cell-specific binding preferences.

### Indirect binding and alternative analyzes

In the above experiments, we used the Unibind database and the PWM associated with the target TF to select ChIP-seq experiments that are unlikely to be affected by technical issues or indirect binding. Moreover, sequences with no binding-motif occurrence around the peak summit were discarded. As we selected only ChIP-seq experiments with good Unibind *p*-values, this involves very few sequences in the 272 experiment (Additional file 1: Fig. S1C). However, in certain cases, peaks without the target motif may also be of interest, especially if indirect binding is suspected. We thus included an option to run TFscope analyses without re-centering the sequences on the most likely TFBS. In this case, the TFscope-DM module is skipped and class prediction is performed using only the nucleotidic environment and the co-factors. As a case study, we selected a new set of 14 experiments with non-significant *p*-values according to Unibind and ran TFscope with and without re-centering on the most likely TFBS of each sequence. As illustrated on Additional file 1: Fig. S5B, in these conditions, better results are obtained on the original sequences, without re-centering on the most likely TFBSs, and without running the TFscope-DM module. However, users should keep in mind that, under such conditions, where the target motif is not enriched in peak sequences, the good performance achieved by TFscope may reflect technical artifacts rather than a real cellular specificity [21]. These results are available on https://gite.lirmm.fr/rromero/tfscope/-/tree/main/results/alternative_analyses/without_motif

Similarly, in the above experiments, we searched for sequence features that may discriminate peaks specific to each cell type. An alternative analysis that could be interesting in certain cases is to identify sequence features differentiating peaks unique to one cell type from peaks common to both cell types. Such an analysis can also be done with TFscope. As an example, we selected the 20 out of 272 experiments with the largest number of common peaks and ran two types of TFscope analyses on these pairs: one to discriminate peaks unique to the first vs. the second experiment (classical analysis) and another one to discriminate the peaks unique to either the first or the second experiment vs. the common peaks (Additional file 1: Fig. S5B). As expected, the second type of analysis appears to be more difficult in terms of AUROC, but still several sequence features partly explain the binding differences, and the AUROC remains high in certain experiments. The results obtained in the 20 experiments are available at https://gite.lirmm.fr/rromero/tfscope/-/tree/main/results/alternative_analyses/inter_comp

Another alternative analysis that can be interesting is to use a dedicated software to select the set of peaks to compare instead of our approach based on peak intersection. Specifically, several differential-peak callers can be used for this purpose (see for example reference [16] for a benchmark of these methods). As a case study, we used the DiffBind method [52] to identify differential peaks on three new experiments comparing ENCODE ChIP-seq peaks of three TFs in different cell types (FOS in IMR90 vs. MCF7, SP1 in HepG2 vs*.* K562, and USF1 in WTC11 vs*.* K562). We ran TFscope on the identified sets of peaks, as well as on the peaks selected with the peak intersection procedure. Results obtained with the two peak selection procedures were very close, with comparable AUCs and similar sequence features identified (see https://gite.lirmm.fr/rromero/tfscope/-/tree/main/results/alternative_analyses/diffbind). Given one ChIP-seq experiment, we then ask if it would be possible to differentiate peaks that are specific to this experiment (i.e., peaks present in this experiment and absent in the other one) from peaks that are present in both experiments but significantly higher in the first one. Such analysis can also be done with TFscope by appropriately selecting the two sets of peaks. This was done on the three ENCODE pairs, using for each comparison the ChIP-seq experiment with the highest number of peaks as reference. Results show that the two peak categories can often be differentiated (AUCs equal to $65\%$, $75\%$, and $>80\%$ on the three experiments), and several sequence features may explain these differences (see above gitlab repository for all details). For example, in FOS MCF7 vs. IMR90 comparison, TFscope found that the binding sites of co-factor ZEB1 are more often present in peaks that are specific to MCF7 (a breast cancer cell line) rather than peaks that are higher in MCF7 but also present in IMR90 (a lung fibroblast cell line), highlighting the recently identified cooperation between ZEB1 and FOS in breast cancer [19]. Similarly, in USF1 K562 vs. WTC11 comparison, TFscope highlights the known cooperation between USF1 and NF-Y to distinguish the two peak categories [11].

### Analysis of binding differences induced by a specific treatment

We next sought to use TFscope to analyze binding differences observed between two ChIP-seq experiments targeting the same cell type but with two different treatments. To avoid any differences linked to technical issues or antibodies, for these analyses, we selected only ChIP-seq pairs derived from the same study. Seventy-nine ChIP-seq pairs were selected (see the “Material and methods” section) and analyzed, involving a total of 21 different TFs and 81 treatments (see Additional file 1: Fig. S6A for statistics about the number of pairs targeting each TF). As for the cell type comparisons, all results obtained in the 79 experiments are available at https://gite.lirmm.fr/rromero/tfscope/-/tree/main/results. We generally obtained similar results as those for the cell type experiments (see the accuracy plot in Additional file 1: Fig. S6B), although the Jaccard distance between treatments is often smaller than between cell types (Additional file 1: Fig. S6C), i.e., two treatments often show more similar binding sites than two cell types. However, for several experiments, there is a clear difference in the binding sites and TFscope indentifies interesting features.

**Fig. 6:**
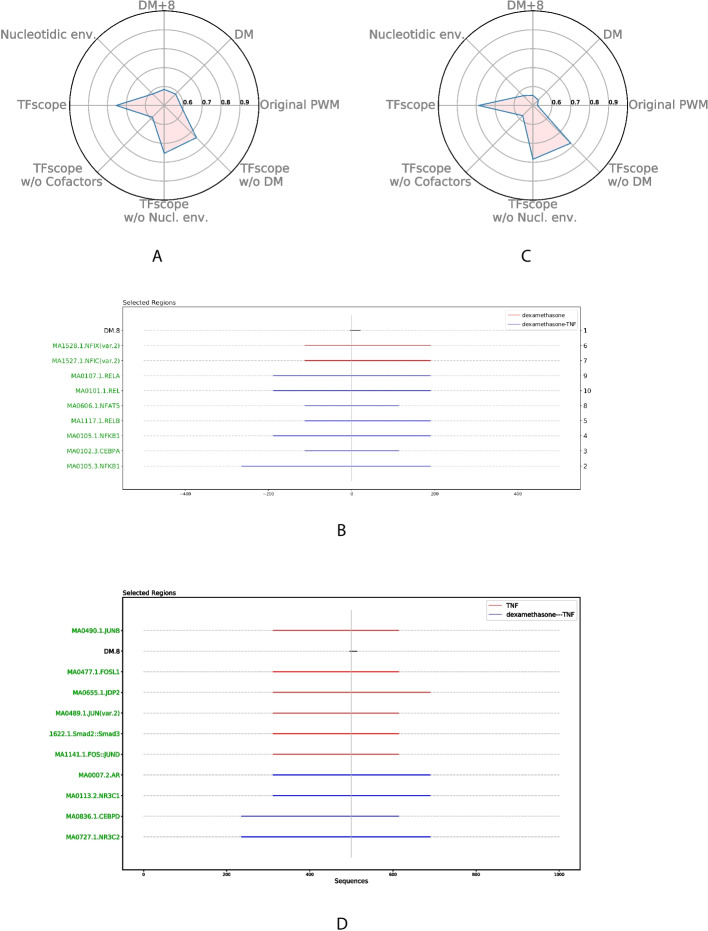
Analysis of binding differences induced by a specific treatment. **A**, **B** Radar plot and location plot of the most important variables in the NR3C1 comparison between Dex and Dex+TNF treatments. **C**, **D** Radar plot and location plot of the most important variables in the RELA comparison between TNF and Dex+TNF treatments

For instance, TFscope confirms the cross-talk between GR signaling and NF-$\kappa$B reported in [29] and proposes additional features. Specifically, analyzing NR3C1 ChIP-seq upon dexamethasone (Dex) and Dex+TNF treatments with TFscope reveals that the main features distinguishing the binding sites in these two conditions are cooperating TFs (Fig. 6A). While motifs of NFI-related TFs are enriched in NR3C1 peaks upon Dex treatment alone, as also observed in [29], motifs of NF-$\kappa$B-related TFs are enriched in NR3C1 peaks upon Dex+TNF (Fig. 6B).

Similarly, cooperating TFs appear to be the main features distinguishing RELA ChIP-seq peaks upon TNF and Dex+TNF treatments (Fig. 6C). TFscope confirms that, in the presence of Dex, RELA peaks are associated with steroid receptor TFs (NR3C1, NR3C2, and AR), but it also suggests that GR signaling diminish cooperation with AP-1 related TFs observed preferentially in RELA peaks in pro-inflammatory conditions (TNF alone) (Fig. 6D).

### Analysis of binding differences of paralogous TFs

**Fig. 7:**
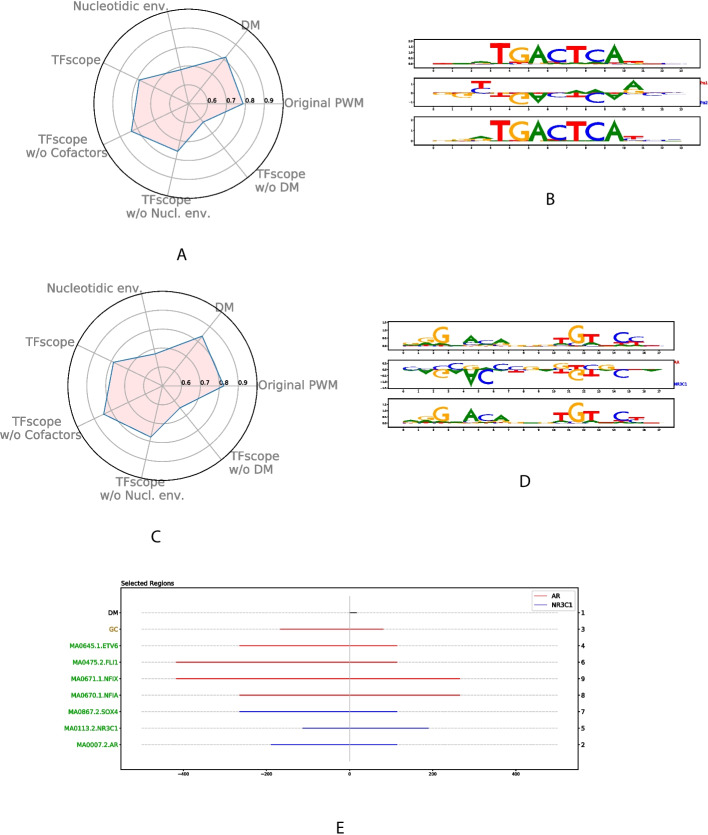
Analysis of binding differences of paralogous TFs. **A** Radar plot of the most important variables discriminating FOSL1 and FOLS2 binding sites. **B** TFscope-DM motif discriminating FOSL1 and FOLS2 binding sites, in between the FOSL1 and FOSL2 PPMs estimated from the same data. **C** Radar plot of the most important variables discriminating AR and GR binding sites. **D** TFscope-DM motif discriminating AR and GR (NR3C1) binding sites, in between the AR and NR3C1 PPMs estimated from the same data. **E** Location plot of variables discriminating AR and GR binding sites

In a previous study [10], we showed that the binding of two paralogous TFs, namely FOSL1 and FOSL2 (also called FRA1 and FRA2), can be distinguished primarily by their motif scores: FOSL2 preferentially binds sequences with high scores for the canonical AP-1 motif, while FOSL1 binds sequences with some degenerate positions (lower scores). We then thought to use TFscope to distinguish FOSL1 from FOSL2 binding in the same dataset. TFscope-DM is indeed sufficient to classify the two peak classes (Fig. 7A) and the typical AP-1 motif is more frequently found in FOSL2 peaks (Fig. 7B), thus confirming our previous results obtained with another approach [56]. Moreover, the discriminative motif also brings new information. For example, FOSL1 favors nucleotides that are inverse from those of the canonical motif at positions 2 and 10.

To confirm the applicability of TFscope for this sort of classification task, we considered another pair of paralogous TFs, i.e., NR3C1 and AR, and ChIP-seq data collected in MCF-7 cells [48]. As shown in Fig. 7C, TFscope is able to accurately distinguish NR3C1 from AR ChIP peaks, and, as for FOSL1/FOSL2, the main differences concern the core motif itself. The output of TFscope-DM reveals that dinucleotides AC at position 4 and GT at position 11 in the canonical NR3C1/AR motif are more frequent for NR3C1 than for AR (Fig. 7D). Moreover, AR ChIP-seq peaks appear to be more GC-rich than NR3C1 peaks (Fig. 7E). These results are in full agreement with those obtained by Kulik et al., who compared AR and GR binding preferences in U2OS cells [35]. Overall, these results illustrate the possibility of using TFscope to distinguish the binding of paralogous TFs.

## Discussion

Here, we proposed a new machine learning approach to identify sequence features that could explain the binding preferences of a TF in two settings: two cell types, two conditions, or two paralogous TFs. Our approach uses three modules that identify three kinds of sequence features related to TF binding. In addition to the specificity of the core motif that is captured by our discriminative PWM, the two other modules extract sequence features related to the nucleotidic environment around the TFBS, and the presence and position of every potential motif of co-factors. A learning algorithm is then run to simultaneously train a model and select the most discriminative features. Hence, contrary to CNN based methods that have been recently proposed, our approach completely controls the predictive features used by the model. This allows us to easily assess the overall importance of each feature, by measuring the loss of accuracy induced by its withdrawal, which would be very challenging to do with CNN approaches.

Our results on different TFs and different cell types show that co-factors are often the most important determinant associated with cell-specific binding sites and that their position relative to the TFBS considered is key. However, for several experiments such as CTCF in CD20 *vs.* RH4, the large nucleotidic environment around the binding sites also partly explains the observed differences. For some other experiments such as JUND in lung vs*.* liver, the main differences directly concern specific binding-site nucleotides. When comparing two treatments, the picture is generally the same, while for paralogous TFs, the main differences are associated with the core motifs in our two experiments. In this latter case, although the binding motifs generally show very similar PWMs for both TFs, subtle differences at specific positions actually explain most of the binding differences.

The first TFscope module captures the features specific to the core motif using a new method to learn a discriminative PWM. Among the numerous approaches already proposed to learn a PWM, it is important to note that most of them actually learn a PPM which is then converted into a PWM with a simple log ratio formula (see for example reference [58]). The problem with this procedure is that it potentially reduces the accuracy of the PWM. Indeed, as PPMs are probabilistic models, they are subject to strong constraints (notably, the sum of a PPM column must be equal to one), which inevitably also constrains the weights of the PWM. For example, the log-ratio operation cannot produce a PWM in which one of the columns has all but one weight equal to zero (the log ratio gives zero when the probability of the nucleotide at this position equals the probability of the nucleotide in the background; but if this is the case for 3 nucleotides it is also necessarily the case for the 4th nucleotide). Interested readers can refer to the work of Ruan and Stormo [45] for further arguments on the limits of PPMs for PWM learning. Our approach based on logistic regression avoids this problem and moreover has the advantage of allowing us to include a LASSO penalty to obtain simpler and more readable PWMs. Another important difference with respect to previous approaches is that in TFscope the discriminative PWM is only used to discriminate the two classes but not to scan the sequences. Hence, TFscope needs two PWMs: the JASPAR PWM is used to scan the sequences and identify the binding sites in both classes, and the discriminative PWM is used to score the binding sites and differentiate the two classes.

### Limits of the study

Our approach could be improved in different ways. Notably, one drawback that can sometimes hamper a straight forward interpretation of the TFscope results is the correlation between predictive variables. Scores of TF motifs especially may be highly correlated, as several TFs often share very similar motifs. Hence, although the PWM highlighted by TFscope is the one that shows the strongest link with the predicted signal, other PWMs could also have a high correlation, and thus other TFs are potential co-factors. We therefore encourage users to refer to PWM clusters as defined, for example, in RSAT-matrix clustering [13]. Similarly, there are sometimes correlations between the nucleotidic composition captured by the TFscope-NE module and the target or co-factor motifs identified by TFscope-CF, especially if the same motif is repeated several times on a sequence. Here again, the linear model and the LASSO penalty ensure that the variables selected by TFscope are those with the strongest link with the predicted signal. Nevertheless, it should be kept in mind that other variables may actually be involved in the studied process. We are thus working on a way to identify and present all alternative variables in a user-friendly interface.

Another clear limit of the approach concerns the incompleteness of the motif library, which can sometimes miss some important binding motifs. In these conditions, the signal may be erroneously attributed to the nucleotidic environment rather than to co-factors or even be completely missed by the approach. Running traditional motif discovery tools [6, 7, 24, 46] and integrating the newly discovered motifs into the motif library before the TFscope analysis could help in such cases.

Finally, it should be noted that other sequence features could be integrated in our model. Specifically concerning co-factors, the number of repeats of a given PWM could be an interesting variable for discriminating two ChIP-seq experiments. Such information is not directly accounted for in the current model but could potentially explain binding differences in some experiments and would avoid the above mentioned confusion between repeated motifs and nucleotide environment.

## Material and methods

### Sequence extraction and alignment

TFscope takes as input two sets of ChIP-seq peaks provided as BED files. First, all peaks common to the two files are removed. This is done with BED tools using
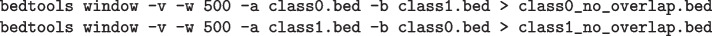


Then, the sequences corresponding to each peak are extracted and aligned on the most likely TFBS occurrence. We thus use the PWM associated with the target TF in JASPAR 2020 [20]. FIMO is used to parse the sequences with the command
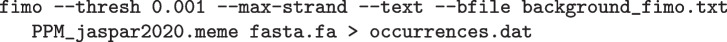


The best occurrence of the motif around the ChIP-seq peak (within a 500-bp limit) is identified and used as an anchor point around which the 1 Kb sequences are centered (see Fig. 1). Sequences for which no occurrence of the motif is found around the peak summit are discarded. Finally, the number of sequences of the two classes are rebalanced, i.e., some sequences of the larger class are randomly selected and removed in order to get two classes with an equal number of sequences. This avoids strong imbalances that may bias parameter estimation in the learning procedure and saves computing time for ChIP-seq experiments with a very large number of peaks. To assess the reproducibility of the results presented here, we measured the variability induced by this re-balancing on 20 randomly selected experiments of different sizes and did not observed large variation in the AUC (Additional File 1: Fig. S8).

When several PWM versions are available in JASPAR, we use the PWM that is the most discriminative for the problem at hand. Namely, for each PWM, the best occurrence of the motif is identified on each sequence, and these scores are used to discriminate the two classes (this corresponds to the AUROC of the original PWM in the radar plots). The PWM version with the highest AUROC is used for the rest of the analysis. Note that composite motifs, i.e., motifs of protein dimers are not considered as a potential version of the motif. The formatted data used in the experiments are available in the dedicated GitLab repository: https://gite.lirmm.fr/rromero/tfscope and on Zenodo[44].

### TFscope-DM

This module takes as input the *K*-length sub-sequence corresponding to the most likely occurrence of the motif in each sequence (with *K* being the size of the PWM). Each sub-sequence *s* is one-hot encoded in a $K\times 4$ matrix **s**. Then, a logistic model with $K\times 4$ parameters (see Expression 2) is learned to discriminate the two classes of sub-sequences. The parameters of the model are estimated by maximum likelihood, with a LASSO penalization [55] to favor simple and easy-to-interpret models. This is done with library glmnet in python 3.

### TFscope-NE

This module takes as input the 1 Kb sequences centered on the most likely TFBS (see the “Sequence extraction and alignment” section). The *K*-length sub-sequence corresponding to the TFBS is masked (replaced by *K* N nucleotides) to avoid capturing information related to the core-motif. Then, the TFscope-NE module constructs new variables defined by a pair (kmer,region) such that the frequency of the identified k-mer in the associated region is, on average, different between the two classes. We thus use a slight modification of the DExTER method [38]: rather than searching for variables that are correlated with an expression signal, TFscope-NE extracts variables that can discriminate the two classes, as measured by the AUROC. The rest of the procedure is exactly the same as that used in DExTER (see ref. [38] for details). Sequences are first segmented into different bins. We used 7 bins in the experiments. TFscope-NE starts with 2-mer (dinucleotides) and, for each 2-mer, identifies the region of consecutive bins for which the 2-mer frequency in the region is the most discriminant. Once the best region has been identified for a 2-mer, TFscope-NE attempts to iteratively extend this 2-mer to identify longer k-mers (up to 4-mers). At the end of the process, a set of variables corresponding to the frequency of the identified k-mers in the identified regions is returned for each sequence.

### TFscope-CF

Like TFscope-NE, this module takes as input the 1 Kb sequences centered on the most likely TFBS (this TFBS is also masked to avoid capturing information related to the core-motif). This module constructs variables defined by a pair (PWM,region) such that the score of the PWM in the identified region is, on average, different between the two classes. For this, sequences are first segmented in bins of the same size. We used 13 bins in our experiments. The number of bins impacts the precision of the approach but also the computing time of the analysis. For each PWM, TFscope scans all sequences with FIMO, and the best score achieved in each bin of each sequence is stored. Then, TFscope uses a lattice structure (see Fig. 1) to compute the best score achieved in any region made up of consecutive bins. Each node of the lattice is associated with a specific region: the top of the lattice represents the whole sequence, while the lowest nodes represent the different bins. Once the best score achieved in every bin has been computed, the best score achieved in any node of the lattice can be easily deduced with a max() operation on its two children nodes. For example, the lattice of Fig. 1 corresponds to a sequence for which the best score is obtained in the first bin ([-500;-300]). For each PWM, a lattice like this one is computed for every sequence. Then, TFscope identifies the node (region) such that the scores associated with this node in the different lattices provide the highest AUROC for discriminating the two classes.

### PPMs associated with each sequence class

In addition to the discriminative PWM learned by the TFscope-DM module, we also estimate two PPMs associated with each sequence class. Given the *K*-length sub-sequences corresponding to the most likely occurrences of the motif in the sequences of one class, a PPM is inferred by simply computing the frequency of each nucleotide at each position. In the output of TFscope, these two PPMs are also provided to help users interpret the discriminative PWM.

Moreover, in the assessment of TFscope, these PPMs were also used to learn a discriminative function that aim to predict if a sub-sequence belongs to the first or the second class. For this, the PPMs are transformed into PWMs and used in the following logistic model:

3 $$\begin{aligned} P(1|s) = S\left( a + b_1 \text {PWM}_1(s) + b_2 \text {PWM}_2(s)\right) , \end{aligned}$$

with *P*(1|*s*) being the probability that sub-sequence *s* belongs to the first class, *S* the sigmoid function, PWM$_1(s)$ and PWM$_2(s)$ the scores of the two PWMs in sub-sequence *s*, and *a*, $b_1$, and $b_2$ the parameter of the logistic model. The accuracy of this approach was compared to that of TFscope-DM using the same sets of training/test sequences (Additional File 1: Fig. S2A).

### Homer analyses

The logistic approach described above was also used with PWMs learned by the Homer approach [24]. We used for this the same *K*-length sub-sequences corresponding to the most likely occurrences of the motif in the two sequence classes, using class 1 and 2 alternatively as forward and backward sequences to get two PWMs, and asking Homer for a motif of length *K*. If more than one motif were returned by Homer in an analysis, the one with the best *p*-value was used. The two PWMs learned on class 1 and class 2 were then integrated into the logistic model of Expression 3 and compared to TFscope-DM in the same set of experiments (see Additional File 1: Fig. S2B). Outputs of the Homer analyses are available on the Gitlab repository at https://gite.lirmm.fr/rromero/tfscope/results/Homer analyzes.

### Selection of 272 ChIP-seq pairs targeting the same TF in two different cell types

Two hundred seventy-two pairs of experiments targeting a common TF, with the same treatment, in two different cell types were selected from the GTRD and UniBind databases. ChIP-seq data were downloaded from GTRD http://gtrd20-06.biouml.org/downloads/20.06/bigBeds/hg38/ChIP-seq/Clusters_by_TF_and_Peakcaller/MACS2/. Only experiments associated with a UniBind *p*-value below $1\%$ were considered, which represents a total of 2815 ChIP-seq data. This data could be arranged in a total of 6553 pairs targeting a common TF with the same treatment in two different cell types. We chose to select only pairs that show highly different peaks for the analyses. This was measured with the Jaccard’s distance. Let A and B be two sets of ChIP-seq peaks on the genome, the Jaccard’s distance $D_J$ is defined from the Jaccard index by:

4 $$\begin{aligned} D_j = 1 - \frac{|A\cap B|}{|A \cup B|}. \end{aligned}$$

Peak intersections and unions were computed with Bedtools window and merge, respectively:
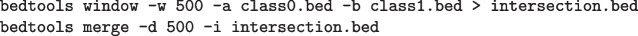


For a given TF and treatment, several pairs with different cell types are often possible. In order to select only a subset of these pairs, we ran a hierarchical clustering of all data targeting the same TF with the same treatment. The clustering was done using the Jaccard’s distance and the complete-linkage agglomeration strategy. We then selected one pair of experiments for each internal node of the tree (the two experiments with the highest number of peaks were selected). Hence, if the tree has *N* leaves (corresponding to the *N* ChIP-seq experiments targeting the same TF and treatment), the number of pairs is exactly *N*. In this way, we end up with a total of 425 ChIP-seq pairs, which were reduced to 368 pairs by selecting only pairs with at least 500 specific peaks in each cell type. Among these pairs, more than 100 actually involved CTCF. We decided to keep only 7 CTCF pairs (which were chosen as the pairs with the largest Jaccard distance), and we end up with a final set of 272 pairs of experiments.

### Measure of PWM simplicity

Information content derived from information theory is often used to measure the conservation of specific nucleotides at specific positions of a motif. This measure is however based on probability distribution and is thus restricted to PPMs: it does not extend to the PWM general case. Hence, we propose to use the Gini coefficient for PWM simplicity measurement.

The Lorenz curve is a graphical representation of the income distribution between individuals in econometrics. It is obtained by ordering the individuals in the order of their income and by calculating the cumulative part of income as a function of the cumulative part of individuals (see Additional File 1: Fig. S7). In the case of an equal distribution between all individuals, the curve follows the line $y=x$. Otherwise, it is found under this line. Surface *A* is the area between the Lorenz curve and the line of perfect distribution equality. Surface *B* is the area between the Lorenz curve and the perfect inequality curve (all the income belongs to a single individual). The Gini coefficient is defined by $A/(A+B)$. It is equal to 1 if all incomes belong to a single individual and is equal to 0 if the incomes are equally distributed.

For PWMs, we compute the Gini coefficient on the set of PWM weights. More precisely, we gather the $4 \times K$ weights of the PWM (all positions combined), order them in ascending order of their absolute value, and compute the Lorenz curve and the Gini coefficient associated with this set of weights. A small Gini coefficient implies an equal PWM weight distribution: the information is dispersed on many PWM elements. On the contrary, a large Gini coefficient (close to 1) indicates that some weights of the PWM gather all the information and that many weights are equal or close to 0. So it can be considered as a measure of model interpretability, where a model with a large Gini coefficient will be simpler than a model with a Gini coefficient close to 0. Importantly, the Gini coefficient does not depend on the scale of the PWM weights but only on their relative importance. Hence, it can be used to compare PWMs obtained through different methods.

### Measure of variable importance

We devised an ad hoc procedure based on LASSO penalty and model error for measuring the individual importance of the different model variables. Given a penalization constraint $\lambda$, the LASSO procedure searches the model parameters that minimize the prediction error subject to the constraint. In practice, a grid of constraints of decreasing values is initialized, and a model is learned for each value. The result is a series of models with an increasing number of parameters. To identify the most important variables of a model in a given condition, we took the model with 10 parameters and estimated the importance of each of the 10 variables in the following way. Given a variable *X*, its importance was estimated by the AUROC difference between the complete model and the model obtained by setting $\beta _X$ at 0.

### Selection of 79 ChIP-seq pairs targeting the same TF with two different treatments

To compare binding preferences upon different treatments, we removed experiments associated with the ‘no-condition’ term in our GTRD/UniBind joint list. We sorted the remaining 1354 experiments according to their GTRD IDs in order to consider experiments from the same publication/study. We further removed time-course experiments and selected 100 pairs of possible comparisons. Then, the same procedure as that used for the selection of the 272 ChIP-seq pairs was applied. This gave a total of 79 pairs of ChIP-seq experiments.

## Supplementary information

Additional file 1. Contains all supplementary figures and tables.

Additional file 2. Peer review history.

## Data Availability

The source code (python), data, and results of the experiments described in this article are available at https://gite.lirmm.fr/rromero/tfscope and on Zenodo [44]. The source code is released under the CeCILL license V2.1.
