# Supplementary material for: TFscope: systematic analysis of the sequence features involved in the binding preferences of transcription factors
Source: Genome Biol. 2024 Jul 10;25:187. doi: 10.1186/s13059-024-03321-8 (PMC11514967; doi:10.1186/s13059-024-03321-8)
Supplement: Supplementary file 1 — Additional file 1. Contains all supplementary figures and tables. [file 13059_2024_3321_MOESM1_ESM.pdf]

# A systematic analysis of the sequence features involved in the binding preferences of transcription factors

## Supplementary Material

Raphaël Roméro[1,2] Christophe Menichelli[1] Christophe Vroland[3,1]
Jean-Michel Marin[2] Sophie Lèbre[2,4†] Charles-Henri Lecellier[3†]
Laurent Bréhélin[1†]

[1] LIRMM, Univ Montpellier, CNRS, Montpellier, France
[2] IMAG, Univ. Montpellier, CNRS, Montpellier, France
[3] Institut de Génétique Moléculaire de Montpellier, University of Montpellier, CNRS, Montpellier, France
[4] Univ. Paul-Valéry-Montpellier, Montpellier, France
[†] Corresponding authors: sophie.lebre@umontpellier.fr, charles.lecellier@igmm.cnrs.fr, brehelin@lirmm.fr

| Repeat class | in_HepG | in_MCF | out_HepG | out_MCF | odds ratio | Fisher pvalue | corr. pvalue |
|---|---|---|---|---|---|---|---|
| DNA | 42 | 30 | 2521 | 2967 | 1.647 | 0.0426 | 0.142 |
| DNA? | 0 | 0 | 2563 | 2997 | nan | 1.0 | 1.0 |
| LINE | 286 | 150 | 2277 | 2847 | 2.383 | 1.815e-17 | 9.078e-17 |
| LTR | 154 | 182 | 2409 | 2815 | 0.988 | 0.954 | 1.0 |
| LTR? | 2 | 2 | 2561 | 2995 | 1.169 | 1.0 | 1.0 |
| Low_complexity | 2 | 151 | 2561 | 2846 | 0.014 | 1.419e-38 | 9.460e-38 |
| RC | 0 | 0 | 2563 | 2997 | nan | 1.0 | 1.0 |
| RC? | 0 | 0 | 2563 | 2997 | nan | 1.0 | 1.0 |
| RNA | 0 | 0 | 2563 | 2997 | nan | 1.0 | 1.0 |
| Retroposon | 3 | 1 | 2560 | 2996 | 3.510 | 0.340 | 0.973 |
| SINE | 97 | 78 | 2466 | 2919 | 1.472 | 0.013 | 0.054 |
| SINE? | 0 | 0 | 2563 | 2997 | nan | 1.0 | 1.0 |
| Satellite | 714 | 4 | 1849 | 2993 | 288.940 | 1.139e-258 | 1.139e-257 |
| Simple_repeat | 58 | 1550 | 2505 | 1447 | 0.0216 | 0.0 | 0.0 |
| Unknown | 0 | 0 | 2563 | 2997 | nan | 1.0 | 1.0 |
| rRNA | 0 | 0 | 2563 | 2997 | nan | 1.0 | 1.0 |
| scRNA | 0 | 0 | 2563 | 2997 | nan | 1.0 | 1.0 |
| snRNA | 0 | 0 | 2563 | 2997 | nan | 1.0 | 1.0 |
| srpRNA | 0 | 0 | 2563 | 2997 | nan | 1.0 | 1.0 |
| tRNA | 0 | 0 | 2563 | 2997 | nan | 1.0 | 1.0 |

**Table S1**: Number of repeats intersecting NRC3C1 ChIP-seq peaks in HepG and MCF10A, for each class of repeats from the RepeatMasker database. Satellites (in MCF10A) and Simple Repeats (in HepG) show the strongest enrichments.

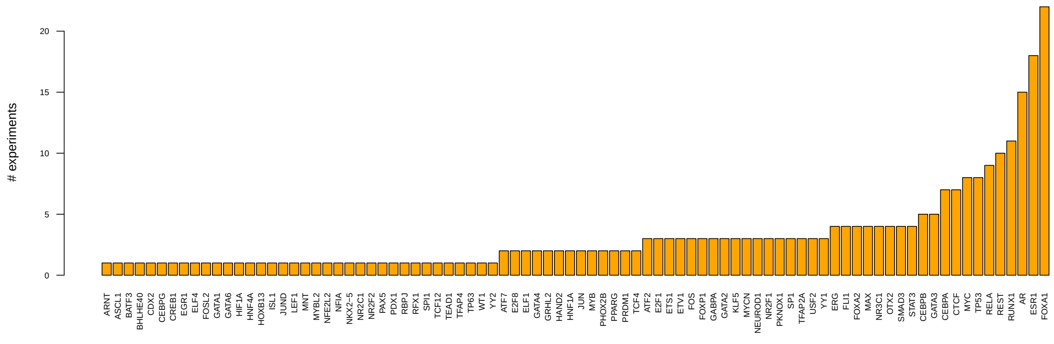


**A**

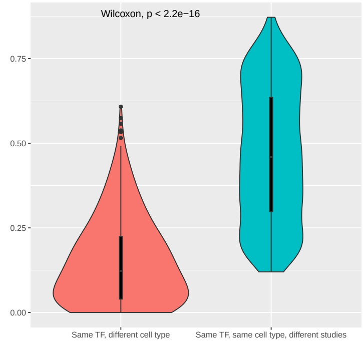


**B**

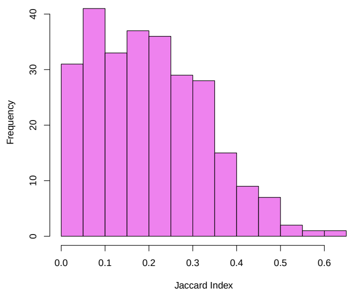


**C**

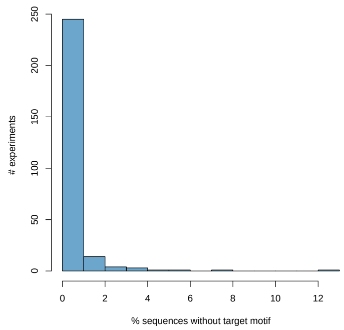


**D**

**Fig. S1**: **Analysis of the cellular specificities of 272 ChIP-seq pairs** **A** Number of experiments targeting each TF in the 272 pairs of ChIP-seq experiments. **B** Distribution of the Jaccard indexes of ChIP-seq pairs targeting the same TF in different cell types (red), and of pairs targeting the same TF in the same cell type but originating from different studies (blue). The Jaccard index measures the proportion of peaks common to two ChIP-seq experiments, with regard to the total number of different peaks in the two experiments, *i.e.* Jaccard index $= \frac{|intersection|}{|union|}$. **C** Distribution of Jaccard indexes of the 272 ChIP-seq pairs. **D** Proportion of sequences without target motif in the 272 experiments. Less than 2% sequences do not have the target motif in most experiments.

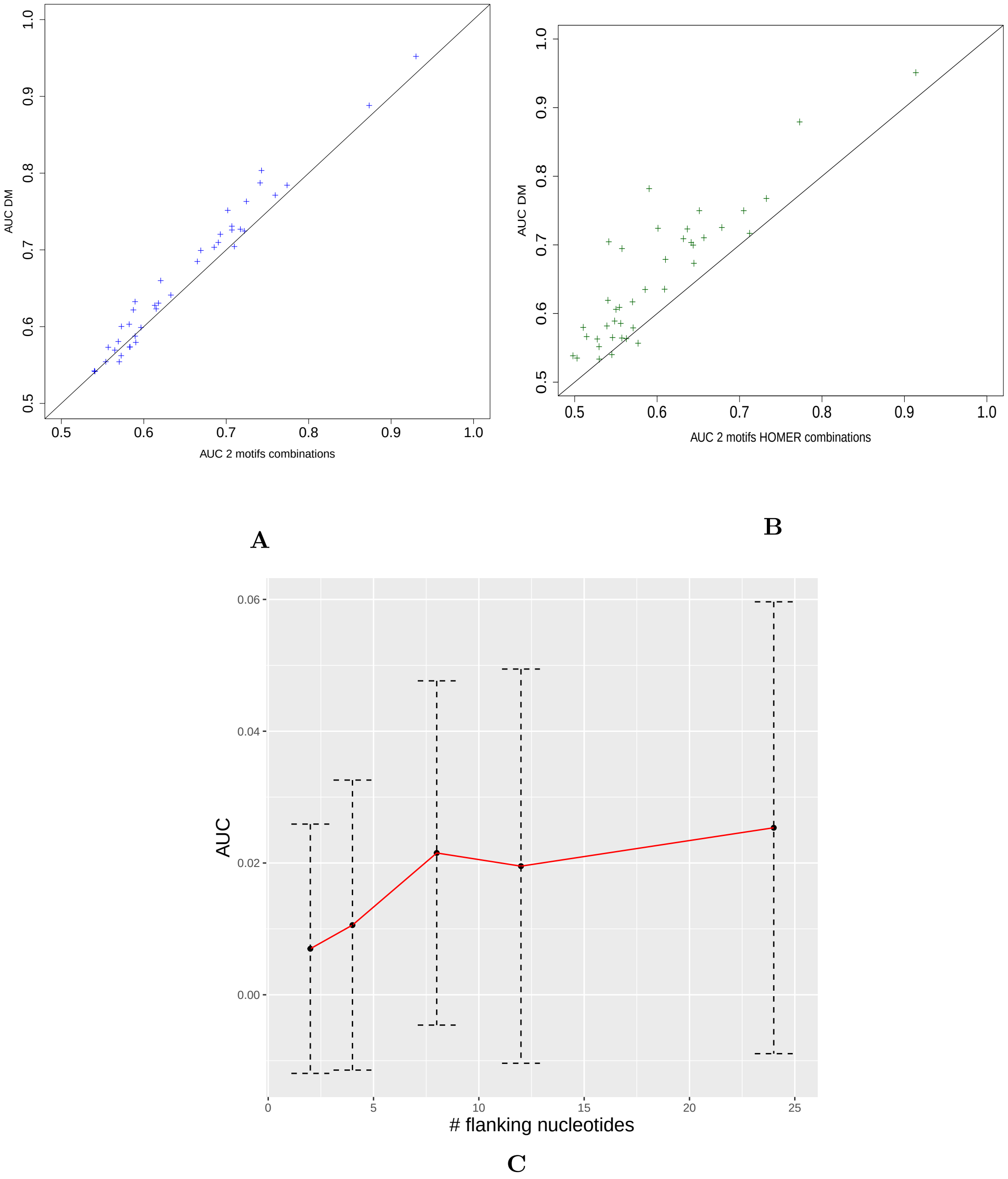


**Fig. S2**: **TFscope learns both discriminative and informative core motifs A** AUROCs achieved by the TFscope PWMs *vs.* the two-PWMs approach on a random selection of 50 pairs of ChIP-seq experiments. **B** AUROCs achieved by the TFscope PWMs *vs.* the two-PWMs approach with PWMs learned by Homer. The same 50 pairs of ChIP-seq experiments as for A were used here. **C** Increasing PWM length improves DM accuracy. The figure reports the difference of AUROC achieved between the DM model with length equal to that of the original JASPAR PWM and models with increasing number of nucleotides on their flanks. Note that the x-axis reports the total number of nucleotides: for example, the 8-nucleotide model has actually 4 nucleotides on both sides.

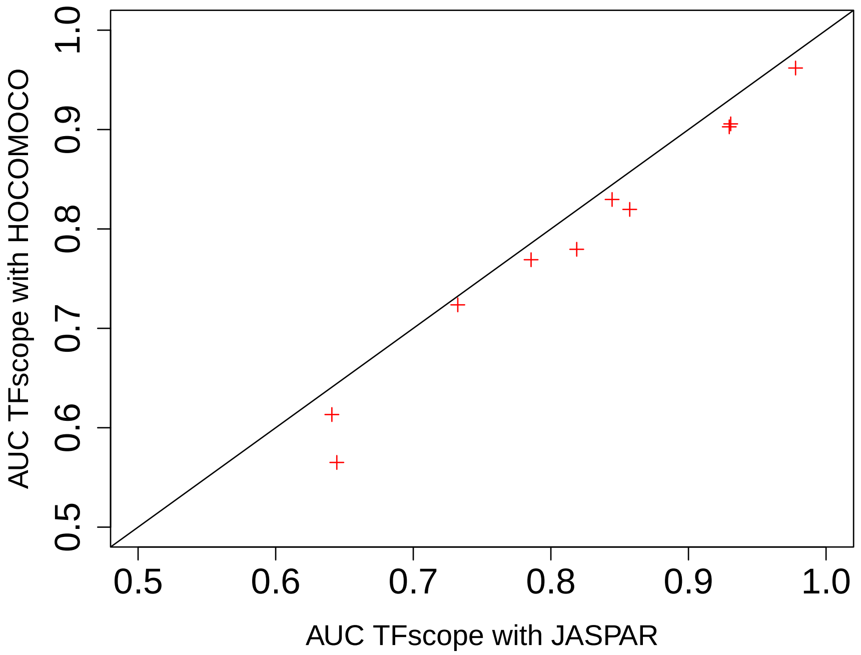


**B**

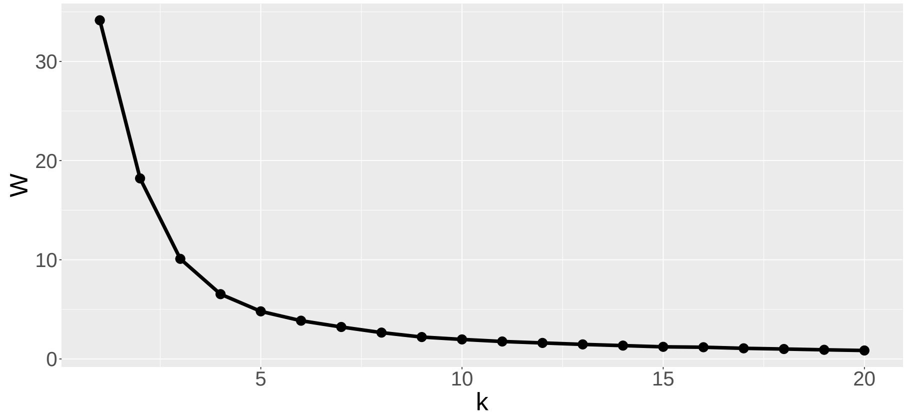


**A**

**Fig. S3**: **TFscope assesses the relative importance of each sequence feature A** Comparison of TFscope AUCs achieved using motifs from JASPAR and HOCOMOCO databases on a random selection of 10 experiments. **B** Within-cluster variance according to the number of clusters present in the 272 importance profiles. Several K-means clustering with different number of clusters were run on the importance profiles inferred by TFscope on the 272 experiments. This Figure plots the total within-cluster sum of squares, that measures cluster scattering, according to the number of clusters. Starting from $k = 1$ (*i.e.* only one cluster), the total within-cluster sum of squares rapidly drops until $k = 3$ or 4, suggesting that only 3 or 4 classes are sufficient to represent most of the points.

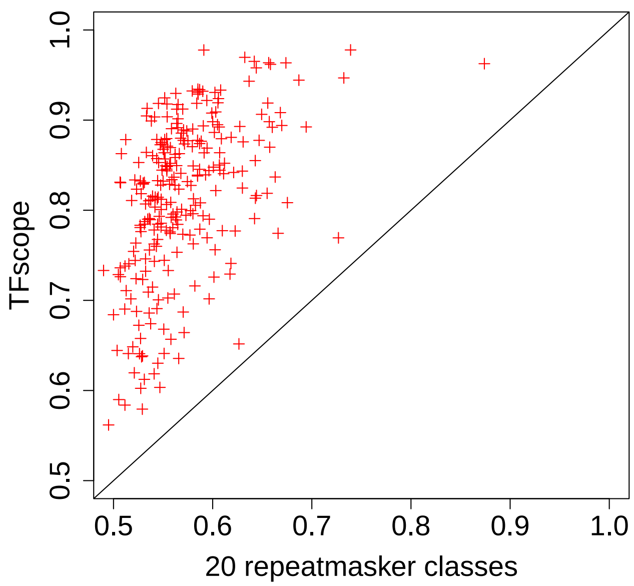


**Fig. S4**: **TFscope correctly handles repeat elements** Comparaison of AUROC achieved by TFscope *vs.* a logistic model that predicts the cell type of a given sequence on the basis of a vector of presence/absence of the 20 repeat classes provided by RepeatMasker.

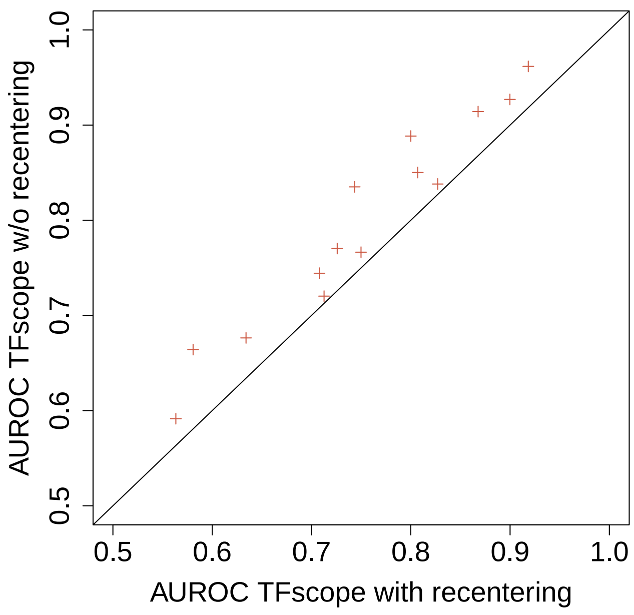


**A**

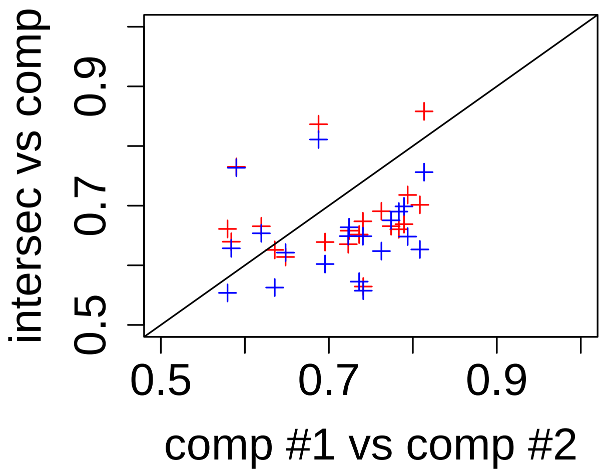


**B**

**Fig. S5**: **Indirect binding and alternative analyses. A** AUROC achieved on 14 pairs of ChIP-seq without good Unibind p-values (*i.e.* without strong enrichment of the target motif in ChIP-seq peaks). The x-axis reports the AUROC achieved with the standard TFscope approach, after re-centering on the most likely TFBS, and removing of the sequences without the target motif. The y-axis is the AUROC achieved by TFscope without removing any sequence, and without re-centering the sequences on their most likely TFBS. **B** This dotplot shows the AUROC achieved on two different discrimination problems for 20 pairs of ChIP-seq experiments comparing one common TF in two different cell types. The x-axis reports the AUROC achieved when comparing peaks unique to the first *vs.* the second cell type. The y-axis reports AUROC achieved when comparing peaks unique either to the first (red points) or the second (blue points) cell type *vs.* all common peaks.

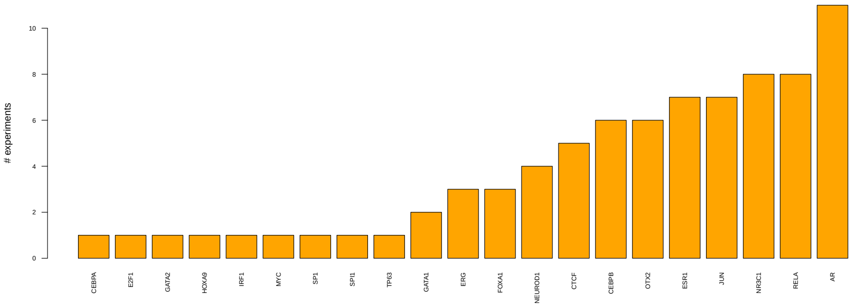


**A**

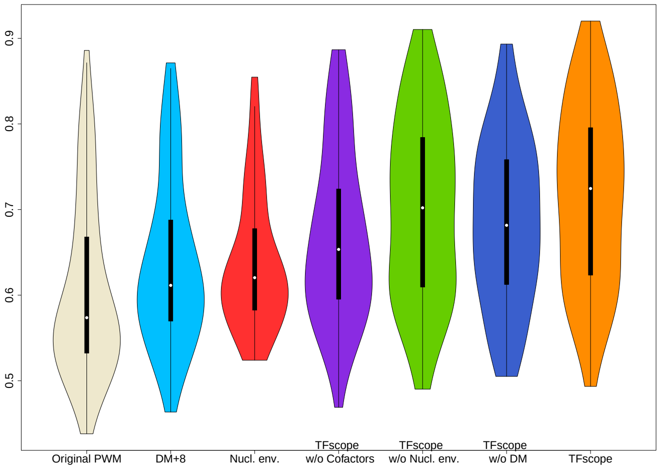


**B**

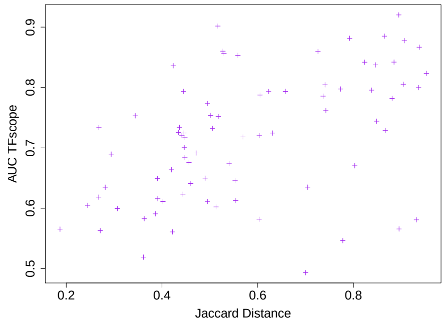


**C**

**Fig. S6**: **Discriminating binding sites of different treatments. A** Number of experiments targeting each TF in the 79 pairs of ChIP-seq experiments. **B** Distribution of AUROCs achieved by TFscope and several alternative models for discriminating binding sites of one TF in two different treatments. **C** Link between TFscope accuracy and the similarity of ChIP-seq peaks in the two treatments. ChIP-seq experiments that have high proportion of peaks in common have low Jaccard distance. Pearson correlation = 0.48

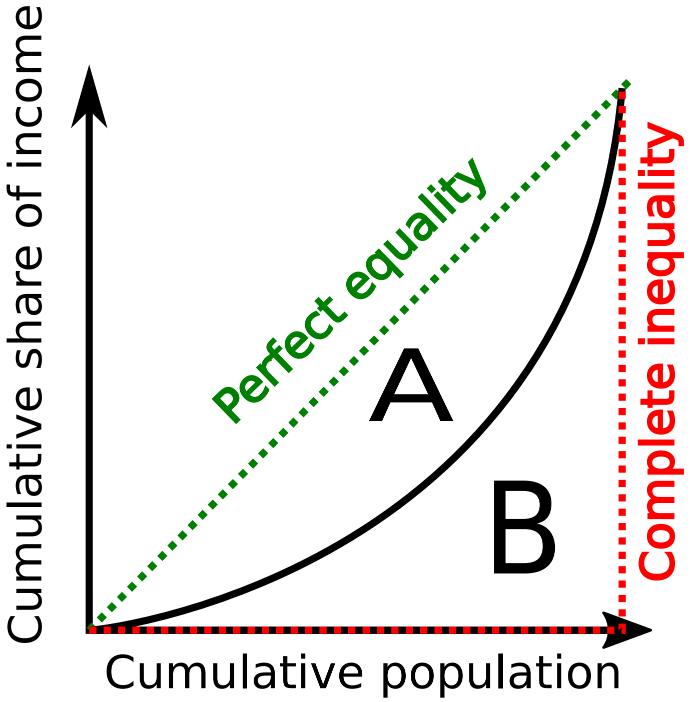


**Fig. S7**: **Lorenz curve and Gini coeficient** Gini coeficient $= A/(A+B)$

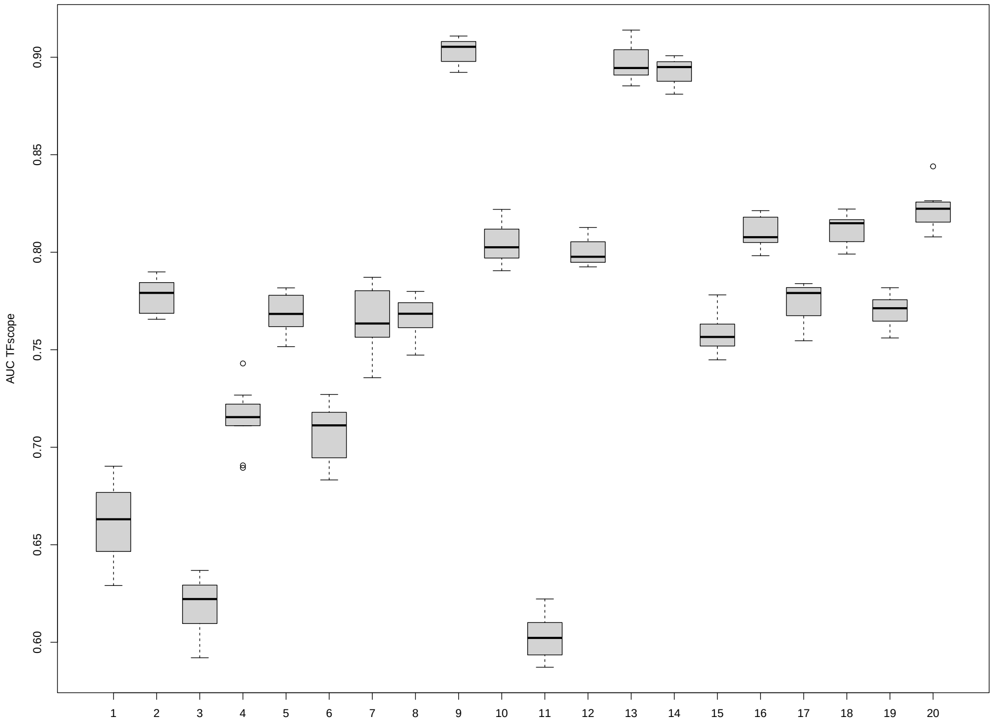


**Fig. S8**: **AUC variation induced by class re-balancing.** 20 experiments among the 272 experiments on cell type specificity were randomly selected. For each experiments, the re-balancing procedure was repeated 10 times (each time the class with the highest number of peaks was randomly sampled to the same number as the class with the smallest number of peaks), and 10 TFscope models were learned. This figure reports the AUC achieved by the 10 models in the 20 experiments ranked from the smallest to the largest number of peaks.
