# Supplementary material for: TFscope: systematic analysis of the sequence features involved in the binding preferences of transcription factors
Source: Genome Biol. 2024 Jul 10;25:187. doi: 10.1186/s13059-024-03321-8 (PMC11514967; doi:10.1186/s13059-024-03321-8)
Supplement: Supplementary file 2 — Additional file 2. Peer review history. [file 13059_2024_3321_MOESM2_ESM.docx]

**Review history**

**First round of review**

**Reviewer 1**

This paper presents a toolkit named TFscope for systemically comparing transcription factor DNA sequence binding preferences between different cell types, experimental conditions, or for paralogous TFs. TFscope was applied to more than 350 pairs of ChIP-seq datasets and was shown to accurately distinguish pairs of TF binding in various settings. The authors presented several showcase examples, including differential binding for JUND in liver vs lung carcinoma, NR3C1 under different treatments of Dex vs Dex+TNF, and comparisons of paralogous TFs (FOSL1 vs FOSL2 and AR vs GR (NR3C1)). The authors assert that co-factors and surrounding nucleotide environment better explain binding differences between different cell types and/or treatment conditions, whereas subtle differences in core motifs seem to be the main reason behind differences observed for paralogous TFs.
Overall, the paper is clearly written and the analyses are largely appropriate. Nevertheless, the following concerns should be addressed prior to publication:

Major concerns:
1. As stated by the authors, the goal of TFscope is "identifying the DNA features explaining the binding differences observed between two ChIP-seq experiments." However, as part of their pre-processing steps, "Sequences with no occurrence of the motif around the peak summit are discarded." Likewise, "To minimize the effects linked to technical issues or indirect binding, data were filtered using the UniBind p-value score" - as stated by the authors, this p-value is largely based on the centrality and strength of the predicted binding site for the ChIP'd TF within the peak.

It is well known that strong binding sites for the ChIP'd TF usually can only be found in about half of ChIP-seq peaks. Some of this could be due to experimental artifacts. But this filter would also miss out on places where the TF is recruited by another TF to the site through a protein-protein interaction. Also, it would miss cases where the TF is binding through an alternative, unknown binding mode (e.g., a C2H2 zinc finger that uses one set of fingers to bind some regions, and another set of fingers to bind other regions). As such, the method is not really doing what the authors claim it is. Instead, it is "identifying the DNA features explaining the binding differences observed between two ChIP-seq experiments WITHIN PEAKS WITH STRONG PREDICTED BINDING SITES." This either needs to be clarified up front and in the discussion, or the procedure needs to be modified to remove this "discarding" step.
2. Likewise, this issue creates problems for the high-level interpretations presented by the authors. Particularly, the authors claim that "for paralogous TFs, subtle differences in the core motif seem to be the main reason for the observed differences in our experiments." However, consider two paralogs (TF_A and TF_B) that primarily interact with TF_X and TF_Y, respectively. As mentioned above, there is a pre-processing step that removes all peaks that lack strong binding sites for TF_A and TF_B. It is quite possible that these peaks would have been highly informative to explain the differences between TF_A and TF_B - namely, the TF_A-specific peaks would have been highly enriched for TF_X binding sites, and the TF_B-specific peaks would have been highly enriched for TF_Y binding sites (e.g., due to physical recruitment of TF_A and TF_B to these sites through TF_X and TF_Y, respectively). However, TFscope would completely miss this because all of these sequences were discarded. I would need to see a new analysis that compares performance of the method with and without this filtering step to see that such is not the case. Or, there needs to be substantial discussion of this topic as a major limitation in the Discussion section, and the authors' need to strongly tone down their assertion that "for paralogous TFs, subtle differences in the core motif seem to be the main reason for the observed differences in our experiments."
3. One of the major concerns regarding the method is its robustness. The authors claimed their method is superior to others by skipping building up the background set. However, it is never shown in the paper what the actual advantages of doing this are. No comparisons are ever performed between the same (or a similar) method that uses such a background. Conceptually, the background (e.g., shared peaks) could indeed provide a good control for marginal features that could get falsely enriched in the differential sets. But this is never explicitly shown.
4. In the methods, it is stated that "The best occurrence of the motif around the ChIP-seq peak (in a limit of 500kb) is identified and used as an anchor point around which the 1Kb sequences are centered." This also seems way too wide. Why would a TF's primary binding site be 500,000 bases away from the peak center? Most ChIP-seq peaks are 200-500 bp wide, which is several orders of magnitude off. This makes me worry that the method is focusing on the completely wrong sites in cases where there are very wide peaks.

Secondary concerns:
1. It is not clear what fraction of peaks are differential across the entire 350 analyzed ChIP-seq pairs under various cell types, treatment conditions, or from a paralogous TF point of view, and whether the background skipping scheme would retain enough statistical power for the downstream TFscope analysis. Put another way, what is the minimum differential peaks needed to properly train and use the model. This would be very useful to know for a user.
2. Fig 2B - it can be hard to understand the "mirror logos" - can the authors also show the two top motifs for the two conditions somehow? Even just showing the initial seed motifs might help. It looks like in SKH1 cells the first half site of the binding site is more or less the same, but the 2nd half-site might be at a different spacing. With the current image, it is really hard to understand what the two motifs really look like on their own (i.e., when not in 'discriminative mode' - what are the actual two motifs bound in the two cell types)?
3. For the second module (TFscope-NE), the authors use a wide window (500bp) around the predicted binding site. This seems to be very wide considering that the goal is to understand the binding of the single TF - 500bp seems much too far for e.g. protein interactions to occur. It would be very useful to see how overall performance is affected when this parameter is varied (e.g., 50bp vs 100bp vs 500bp)
4. The LASSO framework was used to obtain a minimum set of useful features. This tuning process of the penalty multiplier is usually highly data-dependent. Hence, it is not known what the ideal number of features should be targeted by users. The authors should also discuss how these choices would impact outcomes in terms of balancing feature inclusion vs model performance.
5. In the analysis on page 10, the authors should include a k-means plot to show the 4 clusters they claimed to observe in Tfscope's importance profiles. Figure 4C is not be sufficient to support the contention of 4 clusters.
6. The authors should include a plot that summarizes the most important features for all ChIP-seq pairs in all experiments between two cell types, two treatments, and two paralogous TFs. Figures 5, 6, and 7 only focus on one or two particular cases, and an overall pattern of importance scores is never presented.
7. Supplemental Figure 1 shows that DM+8 performs slightly better than DM. It would be interesting to see how large the size of the PWM can be increased until performance is compromised.
8. A prominent general summary of the data used for the study should be included with some information such as all the TFs and cell types, the conditions and treatments of the ChIP-seq data, … Following the same idea from point 1) in the major concern section, the data overview is necessary to determine the significance of the results. How many TF families are covered in this study? How many cell types?

Minor issues
1. "In contrast to the Jun family members, which can homodimerize, the Fos family members must heterodimerize with one of the Jun proteins to bind DNA." Actually, this is no longer thought to be true. At least three different labs have now shown that Fos family members can form homodimers in in vitro systems where Fos is the only TF present:
https://www.ncbi.nlm.nih.gov/pmc/articles/PMC4589601/
https://pubmed.ncbi.nlm.nih.gov/25215497/
https://pubmed.ncbi.nlm.nih.gov/28473536/
2. "DBD motifs as resumed by PWMs" -> should be "represented" or "modeled"
3. "it also suggests that GR signaling abolishes cooperation with AP-1 related TFs" - it does not appear to 'abolish' AP-1 - there are still positive scores associated with those motifs. It seems more like it 'diminishes' it,
4. The method deploys FIMO motif scanning. The best scored motif location is used as an anchoring point for downstream analysis, while discarding peaks without motif instances. This approach should be mentioned in the Discussion as a limitation of the method - the method will not work for TFs without known motifs.
5. TFscope-EN is referenced as a module in Fig. 1, but it is referenced elsewhere as TFscope-NE. This also happened in the Fig. 1 legend.
6. In line 38, the word "explainsmost" needs to be separated.

**Reviewer 2**

The manuscript of Romero et al. presents a new computational approach to identifying sequence-level features that define transcription factor binding differences between cell types, conditions, or close paralogs. The framework includes several clever tricks and builds upon previous tools earlier published by the authors. The manuscript itself is very well-written and describes the approach carefully. Judging by the provided GitHub repo, the accompanying source code is also properly organized and should allow replicating the study and, in theory, running the analysis on user data.

However, there are principal issues that strongly affect the reliability of conclusions and general added value that can be gained by the framework compared to more traditional differential motif discovery.


Major

1. The authors use a fairly straightforward approach (bedtools intersect) to set the ground truth of differential ChIP-Seq peaks between two peak sets. It would be more reliable to apply a proper statistical framework, see a recent benchmark of differential peak calling by Eder & Grebien published in Genome Biol in 2022 (doi: 10.1186/s13059-022-02686-yon).

2. While having a premade workflow or software tool is indeed handy, It is not fully clear what is the key added value of the framework beyond differential motif discovery (which can be done by multiple tools incl. those cited by the authors) and differential motif matching of an existing motif library (which is possible e.g. with HOMER). The authors select the easiest pairs of experiments to discriminate (Page 14, "highly different peaks for analyses") which might be the reason for getting very high classification performance (AUROC). However, I do not see a clear practical application of the classifier per se, except highlighting the key features,
which can be guessed from the alternative existing approaches, even with lower classification AUROC.

3. The authors use a clever approach (TFscope-DM) to build a discriminative model for two sequence sets and show a nice visualization of motif differences. While the technical idea is truly nice, I doubt if it in fact provides a significant improvement over two PWMs (or PFMs) reassembled from motif hits gathered separately from the 0/1 class sequences. I wonder if supplying separate predictions from two such PWMs to the lasso regression would be enough to achieve the same performance.

4. While many subtle differences in TF binding are indeed cell type-specific, the antibodies, and the whole experimental protocol have a significant impact on the outcome, especially, considering non-reproducible peaks (i.e. different between two experiments or replicates). Extra controls are necessary, e.g. explicitly apply TFscope for ChIP-Seq with different antibodies and experimental replicates.

5. The whole approach heavily relies on a staring map of predicted PWM hits. First, reproducing the results with a different (CIS-BP? HOCOMOCO?) motif set might be necessary. Second, on Page 2 the authors mention "a substantial part of the in vivo binding sites lack an obvious match with the known binding motifs of the target TF". Yet on Page 13 "sequence for which no occurrence .. is found .. are discarded" meaning the most non-trivial and substantial set of sequences is discarded from the approach. While running an analysis of the core motif would be hardly impossible for those cases, it is unclear why two other components of the framework were not applied for the motif-less peaks.

6. Linked to the previous point. What is the typical fraction of peaks that can be explained by the authors model? What is the typical fraction of differential peaks that are ignored due to the absence of motif hits? Are the 'shared' peaks a mix or a distinct group in terms of the core motif and/or other features?

7. For any general biological conclusions (as stated in the abstract), it is necessary to perform a systematic analysis across different transcription factor families (i.e. DNA-binding domains).


Minor

1. The authors used UniBind ChIP-Seq tracks. Since they redo the motif analysis, why UniBind was chosen instead of ReMap (https://remap2022.univ-amu.fr/) ?

2. Page 5, "set of sequences that have not been used", more details on the train/test split are necessary.

3. While the authors mention differences in GC content, they do not include (?) these values explicitly in the model. Can these data be used explicitly as a baseline estimate for classification and/or as another feature for the logistic regression?

4. Page 13, "two classes are rebalanced". Why? With any modern ML framework, there should not be any issues in working with disbalances classes.

5. Page 13, "most discriminative PWM from JASPAR" subtypes and composite elements?
For particular TFs, JASPAR includes multiple alternative motif subtypes, e.g. composite elements. It is unclear how it could affect the conclusions, as anything belonging to the starting PWM is attributed to the core motif by the authors, while the motif often resembles not just the binding site but a composite element bound in complex with a common cofactor.

6. Page 10 "the nucleotidic environment around the binding site in 14% cases"; I wonder if this is the case where the incompleteness of the motif library misses some signals which are then erroneously attributed to 'environment' rather than to cofactors. This is the case where differential motif discovery could be more informative.

7. Throughout the text the authors refer to "Genomic features" which usually include genomic tracks from omics experiments. I think 'sequence features' would be a better option.

**Reviewer 3**

SYNOPSIS
The TFscope method is introduced to compare two ChIP-seq datasets in order to identify defining differences between them. It takes as input BAM files describing peak locations, identifies overlapping peaks that are excluded, and focuses subsequent analysis on the two dataset-specific peaks. The comparison of the properties of the two dataset specific peaks includes three major components - the comparison of TF binding motif models, the k-mer composition in the vicinity of the core motif (+/-500bp), and the properties of alternative (cooperative) TF motifs in the vicinity. Results are presented for dataset pairs involving the same TF in different cells and for pairs involving the same cells but different conditions or factors (e.g. TFs sharing a common DBD).

There are a substantial number of matters that need to be addressed, but overall the work is interesting and has the potential to be of high interest to researchers in the field.

MAJOR POINTS
1. The method identifies the overlap between the target sets and excludes the data from the subsequent analysis. This seems to be an important source of information, but there is no assessment presented to explore the consequence of this action or the impact of the parameters used for determining overlap. The results do not seem to convey how much of the datasets fall into this excluded category. There is brief mention that sets with greater difference exhibit different performance properties. Do the property differences observed between the two dataset specific peaks appear in the common peaks? It could be useful to know if one of the two dataset-specific sets is similar in properties to the overlapping set.
2. It was unclear how differences in sources of datasets might impact biological interpretation. Were the sets from different labs? Are there cases where data is available from both one source lab/study and available from different sources? Such data could be used to explore the impact of technical differences, especially for data generated using the same cell lines. While the formatted data are provided in a repository, I recommend that a supplementary table indicating the pairs of datasets be provided as part of the manuscript as it is essential information for reproducibility. Ideally this table would also include links to a resource where it would be possible to determine if the data were from the same or different laboratories. The table could also include some of the information presented visually in figures 2 and 3.
3. The representation of the differences in motif properties between the sets visually highlights the positions of greatest difference. I would also like to understand the significance and scale of differences. Are we visually conveying differences that are actually extremely small? I think each of the depictions should also include the motif model for each set separately to allow a stronger sense of what the similarities are.
4. What is the impact of repetitive sequences? Often in TF analysis we later discover that motif property differences reflect a greater frequency of specific common elements (e.g. retroviruses) in one dataset. Were these sequences masked? If not, a clear analysis of the contribution of such sequence should be provided. What happens if the discriminative model between the datasets is provided with repetitive sequence information (e.g. the presence and absence of the most distinct repetitive elements between the data classes)? As there is the potential that much of the information distinguishing between datasets may relate to such elements, this needs to be explored.
5. The observation that cooperative TF profiles are the most informative seems quite important (and consistent with long history in the field). But the exploration of the meaning of such pairs is handled anecdotally, rather than showing how the cooperative TFs identified relate to biology. Related to the point above, showing the distribution of positions for the strongest cooperative TFs would be helpful to see if there is a strong influence of repetitive sequence.
6. Overall the visualization of the results is quite poor. It is stated in the discussion that future work will describe an interface to the tool. This is fine, but it does not obviate the need to do the best job possible in presenting the results here. In addition to showing the positions of cooperative TF motifs and the dataset specific logos (both indicated above), I recommend that the reports show the number of peaks contributing and the number of overlapping peaks excluded.
7. From the methods… "Finally, the number of sequences of the two classes are rebalanced, i.e. some sequences of the larger class are randomly selected and removed, in order to get two classes with an equal number of sequences." - why do they have to be balanced? What is the impact of this decision? How much data was excluded due to this constraint?
8. The TFSCOPE-NE method only masks the top scoring motif instance for the TF of focus. For many TFs, there is an enrichment of motif occurrence in the vicinity of the strongest motif. What would be the impact of masking all occurrences of the motif over a threshold (or better a range of thresholds) on the results?
9. The selection of datasets required 1000 distinct peaks in each dataset. This limits the analysis to TFs with many peaks (especially after exclusion of overlapping peaks). It would be appropriate to show the distribution of the overlaps to see how this constraint limits the data used.
10. Please report the complete number of times each TF appears in the dataset (CTCF was limited to 7 - does this reflect the maximum that other TFs appear? This could be associated with the requested supplementary table.
11. The discussion section is disappointing. The first paragraph is an unusual mix of summary and specific observations and the rest of the discussion fails to provide perspective about the potential value of the method for the field. I suggest the authors reconsider the discussion and replace it.

MINOR POINTS
1. There are numerous minor language errors. These should be addressed in the editorial process. They do not impede my capacity to understand the research, but addressing them would increase the positive perception of the work.
2. The comparison of FRA-1 and FRA-2 presents the same biological problem as the authors have addressed elsewhere, albeit in a different way. It seems strange to keep coming back to the same pair in different studies. Is there something unique about the pair that makes them work in this context?
3. Vague language "some of the 272" p9-line33 - give the specific number please.
4. "The best occurrence of the motif around the ChIP-seq peak (in a limit of 500kb)" - it seems that the authors meant 500 bp and not 500k

**Authors’ response to reviewers**

Reviewer #1:
This paper presents a toolkit named TFscope for systemically comparing transcription factor DNA sequence binding preferences between different cell types, experimental conditions, or for paralogous TFs. TFscope was applied to more than 350 pairs of ChIP-seq datasets and was shown to accurately distinguish pairs of TF binding in various settings. The authors presented several showcase examples, including differential binding for JUND in
liver vs lung carcinoma, NR3C1 under different treatments of Dex vs Dex+TNF, and comparisons of paralogous TFs (FOSL1 vs FOSL2 and AR vs GR (NR3C1)). The authors assert that co-factors and surrounding nucleotide environment better explain binding differences between different cell types and/or treatment conditions, whereas subtle differences in core motifs seem to be the main reason behind differences observed for paralogous TFs.
Overall, the paper is clearly written and the analyses are largely appropriate. Nevertheless, the following concerns should be addressed prior to publication:

Major concerns:
1. As stated by the authors, the goal of TFscope is "identifying the DNA features explaining the binding differences observed between two ChIP-seq experiments." However, as part of their pre-processing steps, "Sequences with no occurrence of the motif around the peak summit are discarded." Likewise, "To minimize the effects linked to technical issues or indirect binding, data were filtered using the UniBind p-value score" - as stated by the authors, this p-value is largely based on the centrality and strength of the predicted binding site for the ChIP'd TF within the peak.

It is well known that strong binding sites for the ChIP'd TF usually can only be found in about half of ChIP-seq peaks. Some of this could be due to experimental artifacts. But this filter would also miss out on places where the TF is recruited by another TF to the site through a protein-protein interaction. Also, it would miss cases where the TF is binding through an alternative, unknown binding mode (e.g., a C2H2 zinc finger that uses one set of
fingers to bind some regions, and another set of fingers to bind other regions). As such, the method is not really doing what the authors claim it is. Instead, it is "identifying the DNA features explaining the binding differences observed between two ChIP-seq experiments WITHIN PEAKS WITH STRONG PREDICTED BINDING SITES." This either needs to be clarified up front and in the discussion, or the procedure needs to be modified to remove this
"discarding" step.


2. Likewise, this issue creates problems for the high-level interpretations presented by the authors. Particularly, the authors claim that "for paralogous TFs, subtle differences in the core motif seem to be the main reason for the observed differences in our experiments." However, consider two paralogs (TF_A and TF_B) that primarily interact with TF_X and TF_Y, respectively. As mentioned above, there is a pre-processing step that removes all peaks that lack strong binding sites for TF_A and TF_B. It is quite possible that these peaks would have been highly informative to explain the differences between TF_A and TF_B - namely, the TF_A-specific peaks would have been highly enriched for TF_X binding sites, and the TF_B-specific peaks would have been highly enriched for TF_Y binding sites (e.g., due to physical recruitment of TF_A and TF_B to these sites through TF_X and TF_Y, respectively). However, TFscope would completely miss this because all of these sequences were discarded. I would need to see a new analysis that compares performance of the method with and without this filtering step to see that such is not the case. Or, there needs to be substantial discussion of this topic as a major limitation in the Discussion section, and the authors' need to strongly tone down their assertion that "for paralogous TFs, subtle differences in the core motif seem to be the main reason for the observed differences in our experiments."

RESPONSE****************************************************************
Thank you for the positive feedback. We will respond to point #1 and #2 simultaneously as they are highly related (see also our response to Reviewer#2 point #2). UniBind focuses on direct TF-DNA interactions and the selection of ChIP-seq experiments with Unibind p-values < 1e−2 and the removal of sequences without motif is here to avoid, as far as possible, any technical artifacts that would raise concerns about the validity of our analyzes and results. However, we agree with Reviewer #1 that our choice of using UniBind also impedes any analysis of indirect binding. Hence, in this revised version, we added an option to run TFscope without re-centering on the target motif and without removing any sequence (see section « TFscope overview and Figure 1). Moreover, we ran a new set of experiments with this option and showed that TFscope still achieves good accuracy in this condition (section « Indirect binding and alternative analyzes »). Note also that, in this new version, TFscope outputs the number of sequences without motif (see also Supp. Figure 1 for an histogram of this static in our analyzes).


3. One of the major concerns regarding the method is its robustness. The authors claimed their method is superior to others by skipping building up the background set. However, it is never shown in the paper what the actual advantages of doing this are. No comparisons are ever performed between the same (or a similar) method that uses such a background. Conceptually, the background (e.g., shared peaks) could indeed provide a good control for marginal features that could get falsely enriched in the differential sets. But this is never explicitly shown.

RESPONSE****************************************************************
As for the previous points, there is now an option in TFscope to discriminate the peaks unique to one experiment vs. the peaks shared by both experiments (see section “TFscope overview”). Moreover, the new section “Indirect binding and alternative analyses” includes a new set of experiments with this option (see also Supp. Fig. 5B).


4. In the methods, it is stated that "The best occurrence of the motif around the ChIP-seq peak (in a limit of 500kb) is identified and used as an anchor point around which the 1Kb sequences are centered." This also seems way too wide. Why would a TF's primary binding site be 500,000 bases away from the peak center? Most ChIP-seq peaks are 200-500 bp wide, which is several orders of magnitude off. This makes me worry that the method is
focusing on the completely wrong sites in cases where there are very wide peaks.

RESPONSE****************************************************************
This was a regrettable typo that has been corrected. The window is 500bp around the peak and 1kb-long sequences are considered.


Secondary concerns:
1. It is not clear what fraction of peaks are differential across the entire 350 analyzed ChIP-seq pairs under various cell types, treatment conditions, or from a paralogous TF point of view, and whether the background skipping scheme would retain enough statistical power for the downstream TFscope analysis. Put another way, what is the minimum differential peaks needed to properly train and use the model. This would be very useful to know for a user.

RESPONSE****************************************************************
As for any machine learning problem/approach, identifying what is the minimum number of examples (here peaks) required to correctly train a model is not easy, as it strongly depends on the amount of signal in the data. In our opinion, the best approach remains to try and see if the accuracy measured on an independent test set is sufficient, although we can expect that under a few hundred peaks in each class (for example 300), learning the model will certainly be difficult.


2. Fig 2B - it can be hard to understand the "mirror logos" - can the authors also show the two top motifs for the two conditions somehow? Even just showing the initial seed motifs might help. It looks like in SKH1 cells the first half site of the binding site is more or less the same, but the 2nd half-site might be at a different spacing. With the current image, it is really hard to understand what the two motifs really look like on their own (i.e., when not in 'discriminative mode' - what are the actual two motifs bound in the two cell types)?

RESPONSE****************************************************************
We agree that mirror logos can be hard to understand. In this revised version, TFscope outputs the mirror logo along with the traditional motif logos built from the two cell types (see section “TFscope learns both discriminative and informative core motifs”).


3. For the second module (TFscope-NE), the authors use a wide window (500bp) around the predicted binding site. This seems to be very wide considering that the goal is to understand the binding of the single TF - 500bp seems much too far for e.g. protein interactions to occur. It would be very useful to see how overall performance is affected when this parameter is varied (e.g., 50bp vs 100bp vs 500bp)

RESPONSE****************************************************************
500bp is actually the maximum size of the window (more exactly 500bp around the target motif). The TFscope-NE module automatically searches for the best window near (but not necessarily centered on) the motif and, in many cases, the region identified by TFscope is smaller than 100 nucleotides.


4. The LASSO framework was used to obtain a minimum set of useful features. This tuning process of the penalty multiplier is usually highly data-dependent. Hence, it is not known what the ideal number of features should be targeted by users. The authors should also discuss how these choices would impact outcomes in terms of balancing feature inclusion vs model performance.

RESPONSE****************************************************************
The number of features is automatically deduced from the data by the glmnet package. This is done using an internal cross-validation procedure that selects the amount of penalization that maximizes the accuracy of the predictor. This has been clarified in the revised manuscript (section “TFscope overview”).


5. In the analysis on page 10, the authors should include a k-means plot to show the 4 clusters they claimed to observe in Tfscope's importance profiles. Figure 4C is not be sufficient to support the contention of 4 clusters.

RESPONSE****************************************************************
We addressed this point with a new Supp. Figure 3 and editing the end of section “TFscope assesses the relative importance of each genomic feature” p. 9.


6. The authors should include a plot that summarizes the most important features for all ChIP-seq pairs in all experiments between two cell types, two treatments, and two paralogous TFs. Figures 5, 6, and 7 only focus on one or two particular cases, and an overall pattern of importance scores is never presented.

RESPONSE****************************************************************
Figures 4A and 4C and Supp. Fig. 6B summarize the most important features for all experiments between two cell types and two treatments. In addition, the reader can also look at the detailed output of all experiments on the GitLab repository, which provides individual information about the most important features of each analysis. See
https://gite.lirmm.fr/rromero/tfscope/-/blob/main/results/table_discriminating_cell_types.md
https://gite.lirmm.fr/rromero/tfscope/-/blob/main/results/table_discriminating_treatments.md


7. Supplemental Figure 1 shows that DM+8 performs slightly better than DM. It would be interesting to see how large the size of the PWM can be increased until performance is compromised.

RESPONSE****************************************************************
We have conducted the suggested experiment and its results have been included in the revised manuscript (Supp. Fig. 2B and end of section “TFscope learns both discriminative and informative core motifs” p. 7).


8. A prominent general summary of the data used for the study should be included with some information such as all the TFs and cell types, the conditions and treatments of the ChIP-seq data, … Following the same idea from point 1) in the major concern section, the data overview is necessary to determine the significance of the results. How many TF families are covered in this study? How many cell types?

RESPONSE****************************************************************
These statistics have been included in sections “Analysis of the cellular specificities of 272 ChIP-seq pairs” and “Analysis of the binding differences induced by a specific treatment”, and in Supp. Fig. 1. & 6. Moreover, all the analyzes are listed online in two tables that provide links toward the details of the analyzes :
https://gite.lirmm.fr/rromero/tfscope/-/blob/main/results/table_discriminating_cell_types.md
https://gite.lirmm.fr/rromero/tfscope/-/blob/main/results/table_discriminating_treatments.md


Minor issues
1. "In contrast to the Jun family members, which can homodimerize, the Fos family members must heterodimerize with one of the Jun proteins to bind DNA." Actually, this is no longer thought to be true. At least three different labs have now shown that Fos family members can form homodimers in in vitro systems where Fos is the only TF present:
https://www.ncbi.nlm.nih.gov/pmc/articles/PMC4589601/
https://pubmed.ncbi.nlm.nih.gov/25215497/
https://pubmed.ncbi.nlm.nih.gov/28473536/

RESPONSE****************************************************************
We thank the reviewer for pointing out these studies.
Szaloki et al. Mol Cell Biol 2015 concluded that ’Fos homodimerization will be significant only in the case of its selective overexpression’ and that ‘At equal Fos and Jun concentrations, the formation of Fos-Jun heterodimers is more probable than the formation of Fos homodimers’. We have therefore added the reference Szaloki et al. Mol Cell Biol 2015 in the revised manuscript and modified it accordingly (see section “TFscope assesses the relative importance of each genomic feature” p. 9.)
We found no mention of AP-1, FOS or JUN in Weirauch et al., Cell 2014 and Yin et al., Science 2017 but would consider any suggestion from the reviewer.


2. "DBD motifs as resumed by PWMs" -> should be "represented" or "modeled"

RESPONSE****************************************************************
Done


3. "it also suggests that GR signaling abolishes cooperation with AP-1 related TFs" - it does not appear to 'abolish' AP-1 - there are still positive scores associated with those motifs. It seems more like it 'diminishes' it,

RESPONSE****************************************************************
We have modified the manuscript as suggested by the reviewer.


4. The method deploys FIMO motif scanning. The best scored motif location is used as an anchoring point for downstream analysis, while discarding peaks without motif instances. This approach should be mentioned in the Discussion as a limitation of the method - the method will not work for TFs without known motifs.

RESPONSE****************************************************************
This point has been addressed with the new option to run TFscope without target motif. See response to Major concerns 1 & 2.


5. TFscope-EN is referenced as a module in Fig. 1, but it is referenced elsewhere as TFscope-NE. This also happened in the Fig. 1 legend.

RESPONSE****************************************************************
We apologize for this typo, which has been corrected.


6. In line 38, the word "explainsmost" needs to be separated.

RESPONSE****************************************************************
We apologize for this typo, which has been corrected.


Reviewer #2:
The manuscript of Romero et al. presents a new computational approach to identifying sequence-level features that define transcription factor binding differences between cell types, conditions, or close paralogs.
The framework includes several clever tricks and builds upon previous tools earlier published by the authors. The manuscript itself is very well-written and describes the approach carefully. Judging by the provided GitHub repo, the accompanying source code is also properly organized and should allow replicating the study and, in theory, running the analysis on user data.

However, there are principal issues that strongly affect the reliability of conclusions and general added value that can be gained by the framework compared to more traditional differential motif discovery.

Major

1. The authors use a fairly straightforward approach (bedtools intersect) to set the ground truth of differential ChIP-Seq peaks between two peak sets. It would be more reliable to apply a proper statistical framework, see a recent benchmark of differential peak calling by Eder & Grebien published in Genome Biol in 2022 (doi: 10.1186/s13059-022-02686-yon).

RESPONSE****************************************************************
Thank you for this suggestion and the reference. Working on differential peaks is clearly an interesting alternative for certain data/studies. Our approach with bedtools intersect does not answer exactly the same question, as it specifically addresses the peaks that “appear” or “disappear” between two conditions. However TFscope can also be run on peak sets identified with differential peak calling approach. We did it on three examples using the DiffBind method and achieved results similar to that achieved with the bedtools intersect approach in terms of accuracy (see section “Indirect binding and alternative analyzes”). Running these methods requires however to have the bam files of all replicates and conditions, and hence cannot be done at a very large scale from the ChIP-seq databases like GTRD (or ReMap) that we used in our study.


2. While having a premade workflow or software tool is indeed handy, It is not fully clear what is the key added value of the framework beyond differential motif discovery (which can be done by multiple tools incl. those cited by the authors) and differential motif matching of an existing motif library (which is possible e.g. with HOMER). The authors select the easiest pairs of experiments to discriminate (Page 14, "highly different peaks for
analyses") which might be the reason for getting very high classification performance (AUROC). However, I do not see a clear practical application of the classifier per se, except highlighting the key features, which can be guessed from the alternative existing approaches, even with lower classification AUROC.

RESPONSE****************************************************************
The purpose of our selection procedure was not to select the “easiest” pair of experiments to discriminate but to identify pairs that show high differences in their binding sites, because these ones are obviously the most intriguing/interesting cases. Among these pairs, some are easy (i.e. TFscope get high accuracy), but some are not.
In our opinion, the discriminative PWM provided by the TFscope-DM module is interesting in that it directly highlights the key nucleotides that may explain the main differences between the two ChIP-seq. We agree that such information may certainly be deduced using other motif discovery approaches but not in such a direct way. For example, with HOMER or STREME (which both runs one-sided enrichment tests), users would have to run 2 different analyzes by switching the foreground and background sequence sets and then post-process all motifs returned by these analyzes.
Furthermore, we believe that the interest of TFscope is not limited to the TFscope-DM module, but rather lies in its ability to estimate the relative importance of the three kinds of information (target motif, large nucleotidic environment and co-factors). In this perspective, the discriminative PWM returned by TFscope-DM perfectly fits our need, as it can be naturally integrated into the full TFscope model.


3. The authors use a clever approach (TFscope-DM) to build a discriminative model for two sequence sets and show a nice visualization of motif differences. While the technical idea is truly nice, I doubt if it in fact provides a significant improvement over two PWMs (or PFMs) reassembled from motif hits gathered separately from the 0/1 class sequences. I wonder if supplying separate predictions from two such PWMs to the lasso regression would be enough to achieve the same performance.

RESPONSE****************************************************************
Thanks for this suggestion. We included this approach in the revised manuscript and ran some additional experiments to compare its accuracy to that of TFscope-DM (section “TFscope learns both discriminative and informative core motifs” p. 7 and Supp. Fig. 2A). The PWM learned by TFscope outperforms this two-PWMs approach most of the time and especially at AUC > 0.7


4. While many subtle differences in TF binding are indeed cell type-specific, the antibodies, and the whole experimental protocol have a significant impact on the outcome, especially, considering non-reproducible peaks (i.e. different between two experiments or replicates). Extra controls are necessary, e.g. explicitly apply TFscope for ChIP-Seq with different antibodies and experimental replicates.

RESPONSE****************************************************************
First, it is important to note that in all experiments, we selected only ChIP-seq data with Unibind p-values < 1e-2 (i.e. with peaks showing a clear enrichment for the target motif in their center) in order to focus our analyzes on direct TF-DNA interaction and minimize these potential technical artifacts and the problems of antibody specificity (see reference #18).
· Concerning the comparison of pairs of ChIP-seq targeting the same TF with two different treatments, we only selected ChIP-seq pairs originating from the same study/paper, thus controlling potential differences of protocol and antibodies. This point has been clarified in the revised manuscript (section “Analysis of the binding differences induced by a specific treatment” p. 11).
· Concerning the comparisons involving the same TF in different cell types, we have integrated in the revised ms a preliminary statistical analysis that studies the similarity between i) ChIP-seq experiments targeting the same TF in different cell types, and ii) ChIP-seq experiments targeting the same TF in the same cell type but originating from different studies (section “Analysis of the cellular specificities of 272 ChIP-seq pairs” and Supp. Fig. 1B). As shown Supp. Fig. 1B, ChIP-seq pairs from different cell types are usually much more different than experiments in the same cell type. Hence, we concluded that, in these experiments, a large part of the differences observed between cell types likely originates from cellular specificity rather than from technical artifacts.


5. The whole approach heavily relies on a staring map of predicted PWM hits. First, reproducing the results with a different (CIS-BP? HOCOMOCO?) motif set might be necessary.

RESPONSE****************************************************************
As explained above, all the experiments heavily rely on the Unibind quality control, and this database is entirely built on the JASPAR motifs. Moreover, we feel that reproducing the results with different motif databases would lead to compare these databases, which is not the scope of this work.


Second, on Page 2 the authors mention "a substantial part of the in vivo binding sites lack an obvious match with the known binding motifs of the target TF". Yet on Page 13 "sequence for which no occurrence .. is found .. are discarded" meaning the most non-trivial and substantial set of sequences is discarded from the approach. While running an analysis of the core motif would be hardly impossible for those cases, it is unclear why two other components of the framework were not applied for the motif-less peaks.

RESPONSE****************************************************************
Thank you for this suggestion. See also our response to Reviewer#1 point #1. In this revised ms we added an option to run TFscope without re-centering on the target motif and without removing any sequence (see section « TFscope overview and Figure 1). Moreover, we ran an new set of experiments with this option and showed that TFscope still achieves good accuracy in this condition (section « Indirect binding and alternative analyzes »). Note also that in the former analyzes, the number of sequences without the motif is quite low (see new Supp. Fig. 1D), thanks to the use of ChIP-seq data with Unibind p-values < 1e-2.


6. Linked to the previous point. What is the typical fraction of peaks that can be explained by the authors model? What is the typical fraction of differential peaks that are ignored due to the absence of motif hits? Are the 'shared' peaks a mix or a distinct group in terms of the core motif and/or other features?

RESPONSE****************************************************************
See our response to point #5 for the number of peaks without motif. Concerning the shared peaks, we also added an new option that allows TFscope to compare the peaks unique to one ChIP-seq to the peaks shared by the two ChIP-seq (see section “TFscope overview”). Moreover, the new section “Indirect binding and alternative analyzes” includes a new set of experiments with this option. See also our responses to Reviewer#1 point #2 and Reviewer #3 point #1.


7. For any general biological conclusions (as stated in the abstract), it is necessary to perform a systematic analysis across different transcription factor families (i.e. DNA-binding domains).

RESPONSE****************************************************************
We ran a statistical analysis to identify transcription factor families that would behave similarly in term of most important features. Specifically, we searched for enrichment for all potential PFAM DNA binding domains in our clustering of the 272 experiments (Figure 4C), but we did not found any significant enrichment. We are ready to include this negative results if requested by the reviewer.


Minor
1. The authors used UniBind ChIP-Seq tracks. Since they redo the motif analysis, why UniBind was chosen instead of ReMap (https://remap2022.univ-amu.fr/) ?

RESPONSE****************************************************************
Unibind was used to select ChIP-seq experiments that involve direct TF-DNA interactions and are therefore unlikely to be affected by technical artifacts. ReMap has a different purpose. We initially aimed to use ReMap to download the ChIP-seq data used in our analyzes, but it turned-out that there are less links from Unibind to ReMap than from Unibind to GTRD, so we finally used this latter database to download the ChIP-seq data.


2. Page 5, "set of sequences that have not been used", more details on the train/test split are necessary.

RESPONSE****************************************************************
We use 70% sequences for training and 30% for the test. This point has been added in the revised ms.


3. While the authors mention differences in GC content, they do not include (?) these values explicitly in the model. Can these data be used explicitly as a baseline estimate for classification and/or as another feature for the logistic regression?

RESPONSE****************************************************************
The TFscope-EN module is used to capture this type of features. Rather than simply computing this statistic on the whole sequences, it searches for the regions of the sequences that show strongest link between the statistic and their class.


4. Page 13, "two classes are rebalanced". Why? With any modern ML framework, there should not be any issues in working with disbalances classes.

RESPONSE****************************************************************
In our opinion, class imbalance may be a problem for any ML approach (see for example the review below). In our case, re-balancing is just here to avoid strong imbalance during training. When evaluating the accuracy of the approach, this re-balancing has no effect, as the AUROC has the property to be insensitive to this operation, i.e., for a given problem and predictor, the AUROC is exactly the same whatever the class proportions: 50-50%, 20-80%, or even 1-99%.
Johnson, J.M., Khoshgoftaar, T.M. Survey on deep learning with class imbalance. J Big Data 6, 27 (2019). https://doi.org/10.1186/s40537-019-0192-5


5. Page 13, "most discriminative PWM from JASPAR" subtypes and composite elements?
For particular TFs, JASPAR includes multiple alternative motif subtypes, e.g. composite elements. It is unclear how it could affect the conclusions, as anything belonging to the starting PWM is attributed to the core motif by the authors, while the motif often resembles not just the binding site but a composite element bound in complex with a common cofactor.

RESPONSE****************************************************************
The composite motifs (denoted as FOS::JUN for example) are not considered as alternatives for the target motif. However they may appear as co-factors in the analyzes. This point has been clarified in Material and methods, p. 13.


6. Page 10 "the nucleotidic environment around the binding site in 14% cases"; I wonder if this is the case where the incompleteness of the motif library misses some signals which are then erroneously attributed to 'environment' rather than to cofactors. This is the case where differential motif discovery could be more informative.

RESPONSE****************************************************************
We agree with Reviewer #2. This points has been added in the Discussion in a new subsection entitled “Limits of the study”.


7. Throughout the text the authors refer to "Genomic features" which usually include genomic tracks from omics experiments. I think 'sequence features' would be a better option.

RESPONSE****************************************************************
Thank you for this suggestion. This has been modified everywhere in the revised ms., including in the title.


Reviewer #3:
The TFscope method is introduced to compare two ChIP-seq datasets in order to identify defining differences between them. It takes as input BAM files describing peak locations, identifies overlapping peaks that are excluded, and focuses subsequent analysis on the two dataset-specific peaks. The comparison of the properties of the two dataset specific peaks includes three major components - the comparison of TF binding motif models, the k-mer composition in the vicinity of the core motif (+/-500bp), and the properties of alternative (cooperative) TF motifs in the vicinity. Results are presented for dataset pairs involving the same TF in different cells and for pairs involving the same cells but different conditions or factors (e.g. TFs sharing a common DBD).

There are a substantial number of matters that need to be addressed, but overall the work is interesting and has the potential to be of high interest to researchers in the field.

RESPONSE****************************************************************
Thank you for these kind comments.


MAJOR POINTS
1. The method identifies the overlap between the target sets and excludes the data from the subsequent analysis. This seems to be an important source of information, but there is no assessment presented to explore the consequence of this action or the impact of the parameters used for determining overlap. The results do not seem to convey how much of the datasets fall into this excluded category. There is brief mention that sets with
greater difference exhibit different performance properties. Do the property differences observed between the two dataset specific peaks appear in the common peaks? It could be useful to know if one of the two dataset-specific sets is similar in properties to the overlapping set.

RESPONSE****************************************************************
In this revised version TFscope outputs the statistics about the number of unique and shared sequences. Moreover, we have integrated an option to discriminate peaks unique to one experiment vs. peaks shared by both experiments (see section “TFscope overview”). The new section “Indirect binding and alternative analyzes” includes a new set of experiments with this option and compares the results achieved in this setup vs. that of the “classical” analysis that discriminates peaks unique to the first vs. second experiment. See also our response to Reviewer#1 point#2 and Reviewer #2 point #5.


2. It was unclear how differences in sources of datasets might impact biological interpretation. Were the sets from different labs? Are there cases where data is available from both one source lab/study and available from different sources? Such data could be used to explore the impact of technical differences, especially for data generated using the same cell lines. While the formatted data are provided in a repository, I recommend that a
supplementary table indicating the pairs of datasets be provided as part of the manuscript as it is essential information for reproducibility. Ideally this table would also include links to a resource where it would be possible to determine if the data were from the same or different laboratories. The table could also include some of the information presented visually in figures 2 and 3.

RESPONSE****************************************************************
We included two supplementary tables listing the pairs of datasets used in the 272 experiments targeting one TF in two cell types, and the 79 other experiments targeting one TF with two treatments.
https://gite.lirmm.fr/rromero/tfscope/-/blob/main/results/table_discriminating_cell_types.md
https://gite.lirmm.fr/rromero/tfscope/-/blob/main/results/table_discriminating_treatments.md
Concerning the impact different data sources may have on the biological interpretation, the revised manuscript now includes a statistical analysis studying the similarity between i) ChIP-seq experiments targeting the same TF in different cell types and ii) ChIP-seq experiments targeting the same TF in the same cell type but originating from different studies (section “Analysis of the cellular specificities of 272 ChIP-seq pairs” and Supp. Fig. 1B). As shown Supp. Fig. 1B, ChIP-seq pairs from different cell types are usually much more different than experiments in the same cell type. Hence, we concluded that, in these experiments, a large part of the differences observed between cell types likely originates from cellular specificity rather than from technical artifacts.

See also response to Reviewer #2 point #4.

For the 79 comparisons targeting one TF with two treatments, the ChIP-seq data were collected from the same study, so the impact of experimental protocol is supposedly minimal. This point has been clarified in the ms (section “Analysis of the binding differences induced by a specific treatment” p. 11)


3. The representation of the differences in motif properties between the sets visually highlights the positions of greatest difference. I would also like to understand the significance and scale of differences. Are we visually conveying differences that are actually extremely small? I think each of the depictions should also include the motif model for each set separately to allow a stronger sense of what the similarities are.

RESPONSE****************************************************************
We agree that the discriminative motif may be sometimes difficult to understand. Hence, as requested, we included in this revised version the motifs of each set (see for example Figures 2, 5 and 7, and all results presented on the GitLab repository).


4. What is the impact of repetitive sequences? Often in TF analysis we later discover that motif property differences reflect a greater frequency of specific common elements (e.g. retroviruses) in one dataset. Were these sequences masked? If not, a clear analysis of the contribution of such sequence should be provided. What happens if the discriminative model between the datasets is provided with repetitive sequence information (e.g. the
presence and absence of the most distinct repetitive elements between the data classes)? As there is the potential that much of the information distinguishing between datasets may relate to such elements, this needs to be explored.

RESPONSE****************************************************************
Thank you for this suggestion. In the revised ms we evaluated the impact of repetitive sequences on TF binding using the RepeatMasker database, and we compared the results to the output of TFscope. This analysis is described in a new section entitled “TFscope correctly handles repeat elements” p. 9. For most comparisons, AUROC was < 60% (Supp. Fig. 4A), revealing that, in most cases, repeats classes, as opposed to the nucleotidic environment measured by TFscope, play a modest role in discriminating cell-type specific TF binding, with the noticeable example of NR3C1, which is discussed in the revised ms.


5. The observation that cooperative TF profiles are the most informative seems quite important (and consistent with long history in the field). But the exploration of the meaning of such pairs is handled anecdotally, rather than showing how the cooperative TFs identified relate to biology. Related to the point above, showing the distribution of positions for the strongest cooperative TFs would be helpful to see if there is a strong influence of repetitive sequence.

RESPONSE****************************************************************
Thanks for this suggestion. In the revised manuscript, we performed an additional experiment, which shows that the co-factors identified as important by TFscope are often expressed in the cell type where their motifs have the highest score (see section “TFscope provides meaningful information about co-factors” and Figure 3B).


6. Overall the visualization of the results is quite poor. It is stated in the discussion that future work will describe an interface to the tool. This is fine, but it does not obviate the need to do the best job possible in presenting the results here. In addition to showing the positions of cooperative TF motifs and the dataset specific logos (both indicated above), I recommend that the reports show the number of peaks contributing and the number of overlapping peaks excluded.

RESPONSE****************************************************************
All these informations are now provided by TFscope in its output : see all results here
https://gite.lirmm.fr/rromero/tfscope/-/blob/main/results/table_discriminating_cell_types.md
and here
https://gite.lirmm.fr/rromero/tfscope/-/blob/main/results/table_discriminating_treatments.md


7. From the methods… "Finally, the number of sequences of the two classes are rebalanced, i.e. some sequences of the larger class are randomly selected and removed, in order to get two classes with an equal number of sequences." - why do they have to be balanced? What is the impact of this decision? How much data was excluded due to this constraint?

RESPONSE****************************************************************
The re-balancing is done to avoid strong imbalance during training, which may sometimes impact the accuracy of the predictor on the smaller class. As explained to reviewer #2 as well, this re-balancing has no effect on the accuracy estimate of the approach, as the AUROC (the stat. we used to estimate the accuracy) has the property to be insensitive to this operation: for a given problem and predictor, the AUROC is exactly the same whatever the class proportions: 50-50%, 20-80%, or even 1-99%. See also our response to Reviewer #2 minor point #4.


8. The TFSCOPE-NE method only masks the top scoring motif instance for the TF of focus. For many TFs, there is an enrichment of motif occurrence in the vicinity of the strongest motif. What would be the impact of masking all occurrences of the motif over a threshold (or better a range of thresholds) on the results?

RESPONSE****************************************************************
Masking all occurrences of a motif is uneasy, as it requires to define a specific score threshold to decide if a motif is present or not, which may potentially induce masking a lot of positions that are actually not functional binding sites. However we agree that the effect of motifs with numerous repetitions may be incorrectly attributed to the nucleotide environment. This points has been added in Discussion in a new subsection entitled “Limits of the study”. Moreover, to respond to Reviewer#3, we studied the number of times the target motif also appears in the co-factors, which provides a way to identify repeated motifs (see end of section “TFscope assesses the relative importance of each sequence feature”, p. 9). For 32 out of 248 experiments with an AUROC > 70% (13%), the PWM of the target TF is also present among the co-factors, suggesting that the TF often possesses additional binding motifs around the core binding site identified as the most likely TFBS of a sequence, and that these additional binding motifs are more present in one of the two cell types.


9. The selection of datasets required 1000 distinct peaks in each dataset. This limits the analysis to TFs with many peaks (especially after exclusion of overlapping peaks). It would be appropriate to show the distribution of the overlaps to see how this constraint limits the data used.

RESPONSE****************************************************************
In our experiments, we selected datasets with at least 500 distinct peaks in each cell type. This filter limits the data from 425 to 368 pairs (see section “Selection of 272 ChIP-seq pairs targeting the same TF in two different cell types” in Material and methods, p. 15). Note however that this is not a hard limit imposed by TFscope, and a user can choose to analyze datasets with less than 500 sequences in each class. See also our answer to Reviewer #1 secondary concern #1.


10. Please report the complete number of times each TF appears in the dataset (CTCF was limited to 7 - does this reflect the maximum that other TFs appear? This could be associated with the requested supplementary table.

RESPONSE****************************************************************
These statistics have been added in the revised ms (Supp. Figure 1A and Supp. Figure 6A).


11. The discussion section is disappointing. The first paragraph is an unusual mix of summary and specific observations and the rest of the discussion fails to provide perspective about the potential value of the method for the field. I suggest the authors reconsider the discussion and replace it.

RESPONSE****************************************************************
We modified the discussion and included a subsection dedicated to the limits of the study.


MINOR POINTS
1. There are numerous minor language errors. These should be addressed in the editorial process. They do not impede my capacity to understand the research, but addressing them would increase the positive perception of the work.

RESPONSE****************************************************************
The new ms has been corrected by an English native speaker.


2. The comparison of FRA-1 and FRA-2 presents the same biological problem as the authors have addressed elsewhere, albeit in a different way. It seems strange to keep coming back to the same pair in different studies. Is there something unique about the pair that makes them work in this context?

RESPONSE****************************************************************
No, that is just a convenient test to check that TFscope does what is intended for, as we have already worked on this specific problem. Moreover this study allowed us to identify some specific points that were not identified in our previous work, like the inverse nucleotides in positions 2 and 10, that FRA1 seems to accept contrary to FRA2.


3. Vague language "some of the 272" p9-line33 - give the specific number please.

RESPONSE****************************************************************
This has been corrected (“AUROC > 75% for 49 experiments”, p. 7)

4. "The best occurrence of the motif around the ChIP-seq peak (in a limit of 500kb)" - it seems that the authors meant 500 bp and not 500k

RESPONSE****************************************************************
The typo has been corrected.

**Second round of review**

**Reviewer 1**

The authors have done a satisfactory job addressing my concerns in this revision.

**Reviewer 2**

There was a considerable amount of effort invested to improve the manuscript, yet several important points still require further clarification.
Here is the list of key original points that were not fully taken into account while preparing the revised version of the manuscript.

Original major point 1: intersecting the peaks versus applying differential peak calling. While the authors tried to address the question to some degree and report that the method performs adequately on differential peak calls, the principal question still needs to be addressed. The authors claim to study peaks that "appear" and "disappear" between conditions, but it is an oversimplification as the binary peak presence is, by procedure, a matter of selecting particular a peak calling software and thresholds. One would expect there are major "zero-to-nothing" changes as well as minor borderline changes. If the aim is to study the biological phenomena of changed TF binding, one could expect different mechanisms for "altering the peak heights" and "eradicating binding completely". While a complete study, indeed, requires bam alignments, the current case study provides significant room for improvement.

Original major points 2 and 3: the authors have shown that two PWMs per se cannot compete with the differentially constructed matrix, it remains unclear whether the problem lies in rebuilt PWMs and/or scoring mode, and whether the same or better results can be achieved by the existing software. While a comprehensive benchmarking study won't fit the scope of the manuscript, a case study explicitly comparing TFscope-DM against motifs from existing software e.g. HOMER, DREME, Dimont, is necessary to prove the true added value of the approach, instead of just methodological novelty.
In particular, the motif logos presented in the updated manuscript demonstrate minimal differences between experiments in comparison. This raises the question of whether this is truly related to a truly altered binding regime (highly unlike for e.g. CTCF) or some statistical noise that is readily incorporated into the model but cannot be transferred to any other related pair of experiments and thus does not describe any real biology.

Original major point 5: the authors rely on UniBind as a source of annotated ChIP-Seq experiments and JASPAR as a source of PWMs. While this is OK per se, the presented study describes a novel Method. In this regard, either the Method should be considered a supplementary method to the existing databases (UniBind and JASPAR) OR should be proven to work on other data sources.

Original minor point 4. The authors did not answer the question, which was also raised by the other reviewer, and decided to reference the manuscript on class imbalance issues in Deep Learning applications, which is rather irrelevant to this study. I am aware of the applicability of AUROC and similar measures for balanced and imbalanced data. My original question was related to the motivation of balancing the classes at the start of the pipeline, while it can be rigorously done at any later stage. Particularly, it is unclear what fraction of the data was discarded, and whether the results are stable to this arbitrary and probably non-reproducible subsampling procedure. Presenting confidence intervals for performance metrics, and/or quantitative analysis of differences in the resulting diff-PWMs, and/or an overview of what fraction of data was discarded, and/or an estimate of GC balance maintenance during the procedure would be highly beneficial.

**Authors’ response to reviewers**

* Reviewer #2:

There was a considerable amount of effort invested to improve the
manuscript, yet several important points still require further
clarification. Here is the list of key original points that were not
fully taken into account while preparing the revised version of the
manuscript.


* Original major point 1: intersecting the peaks versus applying differential peak calling.

While the authors tried to address the question to some degree and
report that the method performs adequately on differential peak calls,
the principal question still needs to be addressed. The authors claim
to study peaks that "appear" and "disappear" between conditions, but
it is an oversimplification as the binary peak presence is, by
procedure, a matter of selecting particular a peak calling software
and thresholds. One would expect there are major "zero-to-nothing"
changes as well as minor borderline changes. If the aim is to study
the biological phenomena of changed TF binding, one could expect
different mechanisms for "altering the peak heights" and "eradicating
binding completely". While a complete study, indeed, requires bam
alignments, the current case study provides significant room for
improvement.

-- AUTHOR RESPONSE ----------------------------------------------------
Following the reviewer suggestion, we extended the analyzes of
differential peak calling. Specifically, we included a new analysis
investigating the differences between peaks that are specific to a given
ChIP-seq experiment (present in this experiment and absent from the
other one i.e. ‘eradicating binding completely’ mechanism), and peaks
that are present in both experiments but whose intensity is higher in the
first one (i.e. ‘altering the peak heights’ process). We used TFscope for
this classification and showed that these two peak categories can indeed
be also differentiated on the basis of different sequence features. These
analyzes have been included p.11 of the revised ms and have been made
fully available on gitlab:
https://gite.lirmm.fr/rromero/tfscope/-/tree/main/results/alternative_analyses/diffbind
------------------------------------------------------------------------


* Original major points 2 and 3:

The authors have shown that two PWMs per se cannot compete with the
differentially constructed matrix, it remains unclear whether the
problem lies in rebuilt PWMs and/or scoring mode, and whether the same
or better results can be achieved by the existing software. While a
comprehensive benchmarking study won't fit the scope of the
manuscript, a case study explicitly comparing TFscope-DM against
motifs from existing software e.g. HOMER, DREME, Dimont, is necessary
to prove the true added value of the approach, instead of just
methodological novelty. In particular, the motif logos presented in
the updated manuscript demonstrate minimal differences between
experiments in comparison. This raises the question of whether this is
truly related to a truly altered binding regime (highly unlike for
e.g. CTCF) or some statistical noise that is readily incorporated into
the model but cannot be transferred to any other related pair of
experiments and thus does not describe any real biology.


-- AUTHOR RESPONSE ----------------------------------------------------
We totally agree with the reviewer that all binding differences
cannot be attributed to differences in the binding motif identified
with TFscope-DM. The key point of the paper is that these differences
can have different origins (binding motif, presence of co-factor
motifs, or nucleotide environment), and the aim of our approach is to
provide a tool to identify and assess the relative importance of each of
these features. For CTCF, as guessed by the reviewer and as
illustrated on Figure 5.E, the target motif is not relevant to explain
the binding differences because removing this information does not decrease
the accuracy of TFscope in the radar plot. Most of the differences in this
comparison seems rather to originate from the nucleotide environment.

Regarding major point #2, this revised manuscript now includes a comparison
of TFscope-DM against pairs of PWMs inferred with HOMER (findMotifs.pl)
on the same training/test sets. The sequences from the two ChIP-seq
experiments were used as alternatively foreground and background sequences
(see p. 7, Material & Methods p. 15, and Supp. Fig. 2.B). We noticed that the
AUCs obtained with HOMER PWMs are slightly lower than those obtained with
the PWMs estimated from the two sequence sets with a simple "count" approach (see
Supp. Fig. 2A and B). This is likely due to the way HOMER generates its background
and then identifies motifs that appear highly enriched in the foreground set but with
eventually poor discriminative capabilities, an approach that is not the most
appropriate for the task defined in our work.

Finally, we would like to point out that, in our opinion, the best
comparison for TFscope-DM remains the DAMO method, which has been
proposed for very similar purposes, i.e. optimizing a given PWM, in a
discriminative way, and without relying on PPM optimization. Extensive
comparisons in term of AUC and PWM interpretation between TFscope-DM
and DAMO are described in Section "TFscope learns both discriminative
and informative core motifs" p. 7.
------------------------------------------------------------------------

* Original major point 5:

The authors rely on UniBind as a source of annotated ChIP-Seq
experiments and JASPAR as a source of PWMs. While this is OK per se,
the presented study describes a novel Method. In this regard, either
the Method should be considered a supplementary method to the existing
databases (UniBind and JASPAR) OR should be proven to work on other
data sources.

-- AUTHOR RESPONSE ----------------------------------------------------
In this revised manuscript, we also included a new set of experiments done
using PWMs from HOCOMOCO instead of JASPAR. Results are very similar
(see p. 9 and supp. Fig. 3A), indicating that the motif database does not impact
the performance of our approach.

As mentioned in the original ms, UniBind is only used to select high quality ChIP-seq
experiments (as assessed by Unibind p-value). It is not used as the source of
ChIP-seq experiments. Users can of course chose not to use a quality control of their
ChIP-seq experiments.
All BED files were downloaded either from the GTRD database (for the most part) or
directly from ENCODE (for the diffBind experiments). The ChIP-seq data used in all
our experiments originate from many different studies/laboratories. We also show on
supp. Fig. 3A that different motif databases can be used without affecting TFscope
performances. Hence, our method can be used with any data source (ChIP-seq
experiments and motif databases), thereby making it a general approach not
restricted to be used with existing database.

------------------------------------------------------------------------


* Original minor point 4.

The authors did not answer the question, which was also raised by the
other reviewer, and decided to reference the manuscript on class
imbalance issues in Deep Learning applications, which is rather
irrelevant to this study. I am aware of the applicability of AUROC and
similar measures for balanced and imbalanced data. My original
question was related to the motivation of balancing the classes at the
start of the pipeline, while it can be rigorously done at any later
stage. Particularly, it is unclear what fraction of the data was
discarded, and whether the results are stable to this arbitrary and
probably non-reproducible subsampling procedure. Presenting
confidence intervals for performance metrics, and/or quantitative
analysis of differences in the resulting diff-PWMs, and/or an overview
of what fraction of data was discarded, and/or an estimate of GC
balance maintenance during the procedure would be highly beneficial.

-- AUTHOR RESPONSE ----------------------------------------------------
Provided that Deep Learning is considered as ‘modern ML’, we felt that a
reference discussing class imbalance issues in DL was an appropriate response to
the original question of the referee, which was "With any modern ML
framework, there should not be any issues in working with disbalances
classes.".

Following the reviewer’s suggestion, we studied, in this revised manuscript,
the variability of TFscope AUC after 10 re-sampling for 20 different experiments.
Results, presented p. 14 and Supp. Fig. 8, show that this variability is low. We
also examined the GC content of the sequences after each re-sampling. As expected,
GC content is very stable and is not impacted by re-sampling on the same 20
experiments (see Figure “boxplot_stable_gc.pdf” in attached files). At this stage,
we do not believe that the GC stability after re-sampling is an observation that
merits inclusion in the ms but, if the editor thinks otherwise, we will of course abide
by his decision.

Regarding the fraction of discarded data, please note that the number of sequences
before and after class re-balancing have been explicitly indicated in TFscope output,
along with the number of sequences common to both classes, and the proportion of
sequences with the target motif. See all results on gitelab:
https://gite.lirmm.fr/rromero/tfscope/-/tree/main/results
------------------------------------------------------------------------

**Third round of review**

**Reviewer 2**

The authors have made a solid set of revisions addressing the comments and clarifying the issues from the previous review stage.
